# MT-CO2 Dysregulation in Alzheimer’s Disease and Its Modulation by Photobiomodulation: Implications for Mitochondrial Function

**DOI:** 10.3390/cells15211941

**Published:** 2026-10-25

**Authors:** Mohammad Golam Sabbir, Karin Hsiao

**Affiliations:** 1 Alzo Biosciences Inc., San Diego, CA 92121, USA; 2 Capillus LLC, Miami, FL 33131, USA

**Keywords:** Alzheimer’s disease, MT-CO2, cytochrome c oxidase, complex IV, mitochondrial dysfunction, photobiomodulation, near-infrared light, oxidative phosphorylation, neurodegeneration, mitochondrial bioenergetics

## Abstract

**Highlights:**

**What are the main findings?**

- MT-CO2 and SDHB protein abundance are reduced in the temporal cortex of Alzheimer’s disease subjects relative to cognitively normal controls, although the two proteins exhibit distinct diagnostic-group patterns across the examined cohorts.
- Photobiomodulation with 650-nm red light and 808-nm near-infrared light increases MT-CO2 abundance, and shRNA-mediated reduction in MT-CO2 attenuates Complex IV-dependent respiration and photobiomodulation-associated bioenergetic enhancement.

**What are the implications of the main findings?**

- MT-CO2 deficiency may represent a previously underappreciated pathology contributing to mitochondrial dysfunction and impaired oxidative phosphorylation in Alzheimer’s disease.
- Modulation of MT-CO2 by photobiomodulation may represent a potential non-invasive approach for enhancing Complex IV activity and mitochondrial bioenergetics, although its therapeutic relevance to neurodegenerative disease remains to be established.

**Abstract:**

Mitochondrial dysfunction contributes to Alzheimer’s disease (AD), but the roles of individual respiratory-chain proteins remain incompletely understood. We examined mitochondrially encoded cytochrome *c* oxidase subunit II (MT-CO2) and nuclear encoded succinate dehydrogenase complex iron-sulfur subunit B (SDHB) in postmortem temporal cortex from cognitively normal (CN) individuals and patients with AD, Lewy body dementia/Parkinson’s disease (LBD/PD), or progressive supranuclear palsy (PSP). MT-CO2 abundance was lower in AD than in CN and LBD/PD, whereas SDHB was lower in AD than in CN, LBD/PD, and PSP. A significant protein × diagnosis interaction indicated distinct diagnostic-group patterns for MT-CO2 and SDHB. In BE(2)-C neuroblastoma cells, repeated 650 nm red-light and 808 nm near-infrared (NIR) photobiomodulation increased MT-CO2 abundance, with the largest responses following 10- and 15-min exposures. shRNA-mediated MT-CO2 reduction impaired Complex IV-dependent respiration and attenuated the bioenergetic response to 808 nm treatment. Photobiomodulation did not significantly alter mitochondrially encoded MT-CO1 or MT-CO3 abundance. These findings identify alterations in MT-CO2 and SDHB in AD temporal cortex and demonstrate that MT-CO2 contributes to Complex IV-dependent respiration and photobiomodulation-associated bioenergetic enhancement. Because mitochondrial content was not independently measured, the tissue findings cannot distinguish broader mitochondrial loss from protein-specific dysregulation.

## 1. Introduction

Alzheimer’s disease (AD) is the most prevalent neurodegenerative disorder and the leading cause of dementia worldwide [1]. Clinically, AD is characterized by progressive memory loss, cognitive decline, and behavioral disturbances that ultimately impair independent daily functioning [1]. Despite decades of intensive research and significant advances in understanding disease pathology, there is currently no cure for AD [1]. Recent advances in disease-modifying therapies have led to the approval of anti-amyloid monoclonal antibodies for selected patients with early-stage AD [2,3,4]. These therapies reduce amyloid burden and have demonstrated ability to slow cognitive and functional decline, representing an important advance beyond symptomatic treatment [3,5,6,7]. However, their clinical benefits remain modest, and their use may be limited by patient-selection requirements, accessibility, treatment burden, and adverse effects, including amyloid-related imaging abnormalities [8,9,10]. Moreover, these therapies do not halt or reverse disease progression [11]. Therefore, continued investigation of complementary disease mechanisms and therapeutic strategies remains essential.

Several hypotheses have been proposed to explain the pathogenesis of AD, among which the amyloid cascade and tau hypotheses have received the greatest attention [12]. The limitations of current anti-amyloid therapies, however, highlight the need to examine additional biological processes that may contribute to disease initiation and progression [13,14,15,16]. Among these, the “mitochondrial cascade hypothesis” has gained considerable support, proposing that mitochondrial dysfunction is an early and potentially initiating event that may drive amyloid-β accumulation, tau pathology, neuronal dysfunction, and progressive neurodegeneration [17].

Mitochondria are essential organelles responsible for ATP production, calcium homeostasis, redox regulation, apoptosis, and cellular signaling [18]. Neurons are particularly vulnerable to mitochondrial dysfunction because of their exceptionally high energy demands and limited regenerative capacity [19]. Consistent with this notion, numerous studies have reported impaired mitochondrial bioenergetics [20], defective mitophagy [21], increased oxidative damage [22], and mutations in mitochondrial DNA (mtDNA) in AD brains [23]. Mitochondrial dysfunction has also been linked to synaptic failure [24], neuroinflammation [25], and neuronal loss [26,27], all of which contribute to cognitive decline.

The primary function of mitochondria is energy generation through oxidative phosphorylation (OXPHOS), a process mediated by the electron transport chain (ETC) located within the inner mitochondrial membrane. Among the five ETC complexes, Complex IV (cytochrome c oxidase; COX) serves as the terminal enzyme, catalyzing electron transfer from cytochrome c to molecular oxygen and thereby sustaining ATP production. The catalytic core of Complex IV is formed by three mitochondrially encoded subunits, MT-CO1 (Mitochondrially Encoded Cytochrome C Oxidase I), MT-CO2, and MT-CO3. Given its essential role in mitochondrial respiration and neuronal energy metabolism, Complex IV dysfunction has long been considered an important feature of AD-associated mitochondrial impairment [28,29].

Among the mitochondrially encoded Complex IV subunits, MT-CO2 (also known as COX-II) is of particular interest because pathogenic variants in this gene have been associated with mitochondrial disorders characterized by neurodegeneration, neurodegeneration with brain iron accumulation (NBIA) [30], and late-onset cerebellar ataxia [31]. Furthermore, hypermethylation of the MT-CO2 locus accompanied by reduced MT-CO2 expression has been reported in the hippocampus of APP/PS1 transgenic mice [32]. These observations suggest that MT-CO2 may play an important role in maintaining neuronal mitochondrial function and raise the possibility that altered MT-CO2 expression contributes to AD-associated mitochondrial dysfunction. Nevertheless, the expression and pathological significance of MT-CO2 in human AD brain tissue remain poorly understood.

Photobiomodulation (PBM), previously known as low-level light therapy, has emerged as a promising non-invasive strategy for modulating mitochondrial function [33,34,35]. PBM utilizes red and near-infrared wavelengths within the wavelength range of approximately 650–1200 nm, that penetrate biological tissues and induce photochemical responses within cells [36]. Although the precise primary photoreceptor remains a subject of debate, COX is widely considered a major mitochondrial target of PBM [37,38]. Light absorption is proposed to enhance electron transport, increase ATP production, improve mitochondrial membrane potential, and stimulate redox-sensitive signaling pathways involved in cellular metabolism, neuroplasticity, and inflammatory regulation [39,40]. Consequently, PBM has gained considerable interest as a potential therapeutic approach for neurodegenerative and other disorders characterized by mitochondrial dysfunction, including AD [41,42,43,44,45,46,47,48]. Despite the growing acceptance of COX as a key mediator of PBM effects, it remains unclear whether PBM influences the expression of individual Complex IV subunits or alters the composition and function of the Complex IV holoenzyme. Understanding how PBM regulates individual Complex IV components is therefore important for elucidating its molecular mechanism of action.

In the present study, we investigated MT-CO2 protein abundance in temporal cortical tissues obtained from postmortem AD patients, cognitively normal (CN) individuals, Lewy body dementia/Parkinson’s disease (LBD/PD) patients, and progressive supranuclear palsy (PSP) patients. Inclusion of the LBD/PD and PSP cohorts enabled exploratory comparisons of MT-CO2 abundance across neurodegenerative disease groups. To assess whether the diagnostic-group pattern was restricted to MT-CO2 or accompanied by changes in another OXPHOS protein detected by the same antibody cocktail, we also quantified SDHB, a nuclear-encoded component of respiratory-chain Complex II. In parallel, we examined the effects of 650 nm and 808 nm light-induced PBM on the abundance of the mitochondrially encoded Complex IV subunits MT-CO1, MT-CO2, and MT-CO3, as well as cytochrome c, somatic (CYCS), in BE(2)-C neuroblastoma cells. Furthermore, using shRNA-mediated gene silencing and cellular bioenergetic analyses, we evaluated the functional contribution of MT-CO2 to PBM-mediated regulation of mitochondrial respiration.

Through these complementary approaches, we sought to establish whether dysregulation of MT-CO2 is associated with AD-related mitochondrial pathology, whether its diagnostic-group pattern differs from that of SDHB, and whether PBM can modulate MT-CO2 expression and Complex IV function. Collectively, these studies examine MT-CO2 and SDHB abundance across neurodegenerative disease groups and investigate the contribution of MT-CO2 to PBM-associated bioenergetic responses in a neuroblastoma cell model.

## 2. Materials and Methods

### 2.1. Human Brain Tissue Samples

Frozen (−80 °C) temporal cortex tissues from cognitively normal CN individuals (*n* = 9), AD patients (*n* = 74), LBD/PD patients (*n* = 38), and PSP patients (*n* = 11) were obtained from the University of Florida Neuromedicine Human Brain and Tissue Bank (Florida, USA). This cohort has been extensively characterized and used previously in studies examining the involvement of Cholinergic Receptor Muscarinic 1 (CHRM1), Calcium/Calmodulin-Dependent Protein Kinase Kinase 2 (CAMKK2), transferrin (TF), and transferrin receptor (TFRC) proteins in AD and other neurodegenerative diseases [49,50]. Detailed demographic and clinicopathological information, including age at death, sex, postmortem interval, and neuropathological classification, is available in the supplementary tables of our previous publication [50]. All tissue samples were stored at −80 °C until further analysis.

### 2.2. Cell Lines and Cell Culture

The BE(2)-C (Catalog No. CRL-2268) neuroblastoma cell line was purchased from the American Type Culture Collection (ATCC) [51]. The BE(2)-C cell line was originally established from the bone marrow metastasis of a male patient with neuroblastoma and is widely used as an in vitro model in neuroscience and neurodegenerative disease research [52]. Cells were cultivated in a base medium consisting of a 1:1 mixture of ATCC-formulated Eagle’s Minimum Essential Medium (ATCC, Manassas, VA, USA; Catalog No. 30-2003) and F-12 Medium (Thermo Fisher Scientific, Waltham, MA, USA; Catalog No. 11765054), supplemented with 10% fetal bovine serum and 1× Antibiotic-Antimycotic solution (Sigma-Aldrich, St. Louis, MO, USA; Catalog No. A5955). Cells were maintained in a humidified incubator at 37 °C under an atmosphere of 5% CO_2_ and approximately 95% relative humidity, and the culture medium was replaced every 2 to 3 days.

### 2.3. Light Treatment

For PBM experiments, exponentially growing cells were seeded in 48-well tissue culture plates and maintained under standard culture conditions. During illumination, culture plates were covered with their lids and irradiated from below. Light was delivered using 650 nm and 808 nm laser diodes powered by a portable power supply unit (Capillus, Miami, FL, USA). Cells were exposed to either 650 nm red light or 808 nm near-infrared (NIR) light at an irradiance of ~5 mW/cm^2^. The distance between the light source and the bottom of the culture plate was maintained at 2 cm, producing a beam diameter of 11 ± 1 mm, which was sufficient to cover the growth area of individual wells in the 48-well plates. To characterize the exposure-duration response and identify the most effective treatment condition, cells were irradiated with either 650 nm red light or 808 nm NIR light for 2, 5, 10, 15, 20, or 30 min per exposure. Each exposure-duration condition was administered every 4 h over a total treatment period of 48 h, resulting in 12 exposures per treatment group. At an irradiance of approximately ~5 mW/cm^2^, the 2-, 5-, 10-, 15-, 20-, and 30-min treatments delivered approximately 0.6, 1.5, 3.0, 4.5, 6.0, and 9.0 J/cm^2^ per exposure, respectively. The corresponding cumulative fluences delivered over the 12 exposures were approximately 7.2, 18, 36, 54, 72, and 108 J/cm^2^, respectively. No-light control cells were maintained under otherwise identical culture conditions without irradiation. All experiments were performed with multiple biological replicates and compared with no-light control groups maintained under identical culture conditions. Following completion of the 48-h treatment regimen, cells were either harvested directly or pooled from replicate wells, as appropriate, for subsequent protein expression and bioenergetic analyses.

### 2.4. Western Blotting

Postmortem temporal cortex tissues and BE(2)-C cells were lysed in phosphate-buffered saline (PBS; 137 mM NaCl, 2.7 mM KCl, 10 mM Na_2_HPO_4_, and 1.8 mM KH_2_PO_4_; pH 7.4) supplemented with 1% NP-40 and protease and phosphatase inhibitor cocktails. Protein concentrations were determined using the Bradford assay. Depending on the molecular mass of the target protein, 50 μg of total protein from each sample was separated on 10–15% SDS-polyacrylamide gels.

For each immunoblot experiment, aliquots of the same protein lysates were loaded in corresponding lanes of multiple gels, which were electrophoresed in parallel under identical conditions. One gel was stained with Oriole (Bio-Rad Laboratories, Hercules, CA, USA) fluorescent gel stain to determine the total-protein intensity in each lane. Proteins resolved on the corresponding parallel gel were transferred to a 0.2-μm nitrocellulose membrane using the Bio-Rad Trans-Blot Turbo Transfer System and used for immunodetection of the proteins of interest. Because the paired gels contained aliquots of the same lysates arranged in the same lane order and were electrophoresed under identical conditions, the Oriole-stained total-protein intensity in each lane was used to normalize the intensity of the corresponding immunoreactive band. This parallel-gel total-protein normalization strategy has been validated and described in detail in our previous studies [50,53].

Membranes were blocked with 5% nonfat dry milk prepared in PBS containing 0.1% Tween 20 and incubated with the indicated primary antibodies. For OXPHOS immunoblotting, membranes were incubated with the Total OXPHOS Human Western Blot Antibody Cocktail (Abcam, Cambridge, UK; Catalog No. ab110411; dilution 1:1000). This premixed reagent contains antibodies recognizing NDUFB8, SDHB, UQCRC2, MT-CO2, and ATP5A, representing mitochondrial respiratory-chain Complexes I–V, respectively. Additional immunoblots were performed using primary antibodies against MT-CO1(Cell Signaling Technology, Danvers, MA, USA; Catalog No. 55159; dilution 1:2000; supplier-reported endogenous molecular weight, approximately 32 kDa), MT-CO2 (Abcam, Cambridge, UK; catalog no. ab110258; dilution 1:2000; supplier-reported endogenous molecular weight, approximately 25 kDa), MT-CO3 (Novus Biologicals, Centennial, CO, USA; Catalog No. NBP2-92765; dilution 1:2000; supplier-reported endogenous molecular weight, approximately 25 kDa), CYCS (Cell Signaling Technology, Danvers, MA, USA; clone D18C7; Catalog No. 11940; dilution 1:3000; supplier-reported endogenous molecular weight, approximately 14 kDa), ATP5A (Abcam, Cambridge, UK; Catalog No. ab14748; dilution 1:3000; supplier-reported endogenous molecular weight, approximately 50 kDa), and GAPDH (Cell Signaling Technology, Danvers, MA, USA; Catalog No. 2118; dilution 1:3000; supplier-reported endogenous molecular weight, approximately 37 kDa). Following incubation with the appropriate horseradish peroxidase-conjugated secondary antibodies, immunoreactive signals were detected using a LICOR Odyssey XF Imaging System (LI-COR Biosciences, Lincoln, NE, USA).

Immunoblot images were quantified using ImageJ software (version 1.48v). The protein-loading and image-acquisition conditions used for densitometry were established during assay optimization to maintain the measured immunoreactive signals within the quantitative range. Chemiluminescent images were acquired at multiple exposure times using the cumulative acquisition mode of the LI-COR Odyssey XF Imaging System. For each target protein, an exposure at which the immunoreactive bands remained unsaturated and within the quantitative detection range was selected for densitometric analysis; saturated exposures were not used for quantification. Local background signal was subtracted from the integrated intensity of each target-protein band.

Because the primary objective of the present study was to examine MT-CO2 abundance, the OXPHOS-cocktail immunoblots were evaluated primarily for the MT-CO2-specific signal. To determine whether the diagnostic-group pattern observed for MT-CO2 was accompanied by changes in another OXPHOS protein detected on the same immunoblots, the SDHB signal was subsequently quantified. SDHB was selected because it produced a distinct signal that could be consistently quantified under the image-acquisition conditions used in the human-tissue experiments. The remaining cocktail-derived signals were used to support band identification but were not included in the human-tissue quantitative analysis. MT-CO2 was identified according to its electrophoretic migration relative to the molecular-weight standards and the other cocktail-derived signals. The NDUFB8 immunoreactive signal migrated immediately below MT-CO2 and was weaker under the protein-loading and image-acquisition conditions used for MT-CO2 quantification. MT-CO2 and SDHB densitometry was performed using the Gel Analyzer function in ImageJ. Rectangular selections of consistent width were applied to individual lanes, and the resulting intensity profiles were used to distinguish the MT-CO2 peak from the immediately lower-migrating NDUFB8 signal. The NDUFB8-associated component was excluded, and the background-corrected MT-CO2 peak area was measured using the Wand (Tracing) tool. Only nonsaturated images in which the two signals could be adequately distinguished were used for quantification. Higher-exposure images were used only to verify the positions of weaker cocktail-derived signals and were not used for MT-CO2 densitometry. A representative example of this procedure is provided in Supplementary Figure S1.

For postmortem temporal cortex samples, the background-corrected intensity of each MT-CO2 or SDHB band was normalized to the corresponding Oriole-stained total-protein intensity. The CN sample A21-006 was included on every gel as a common inter-blot reference. Following normalization for protein loading, the value obtained for A21-006 was assigned as 100%, and the relative abundance of each target protein was calculated as follows: Relative protein abundance (%) = (normalized band intensity of the sample/normalized band intensity of A21-006) × 100. This procedure corrected for differences in protein loading and enabled quantitative comparison of samples analyzed on separate immunoblots [49,50,54]. The other OXPHOS-cocktail-derived signals, including NDUFB8, UQCRC2, and ATP5A, were not included in the human-tissue quantitative analysis.

For cell-culture immunoblot analyses, background-corrected target-protein band intensities were normalized to the corresponding reference-protein signal. For experiments performed using the OXPHOS antibody cocktail, MT-CO2 intensity was normalized to the ATP5A signal detected on the same cocktail-probed membrane. MT-CO1, MT-CO3, and CYCS band intensities were normalized to GAPDH. For analyses using GAPDH as the reference protein, GAPDH was detected on separate membranes prepared using aliquots of the same lysates arranged in the corresponding lane order. When aliquots of the same lysate were loaded in multiple lanes, these lanes were treated as technical replicates rather than independent observations. The normalized technical-replicate values were averaged within each treatment condition and independent experimental run to generate one experiment-level mean per condition. The broader quantitative immunoblotting framework, including establishment of the appropriate protein-loading range, normalization procedures, inter-blot reference normalization where applicable, and densitometric analysis, has been described in detail in our previous publication [50].

### 2.5. Stable MT-CO2 Knockdown in BE(2)-C Cells

BE(2)-C neuroblastoma cells were transduced with lentiviral particles encoding short hairpin RNAs (shRNAs) targeting the human *MT-CO2* gene. Two independent *MT-CO2*-targeting shRNA sequences, designated shMT-CO2#1 (5′-GAGCAAACCACAGTTTCATGC-3′) and shMT-CO2#2 (5′-GAGTAGTACTCCCGATTGAAG-3′), were cloned into the pLKO.1-puromycin lentiviral vector as previously described [55]. A scrambled shRNA sequence (5′-CCTAAGGTTAAGTCGCCCTCG-3′), with no known homology to human genes, was used as a negative control [56]. Following transduction, cells were allowed to recover for 24 h before selection with puromycin (2 μg/mL) to establish stable knockdown populations. Puromycin-containing medium was replaced every 48 h, and selection was continued for 4 days. At the end of the selection period, cells were harvested, and total protein lysates were prepared. Knockdown efficiency was assessed by Western blot analysis of MT-CO2 protein expression relative to scrambled shRNA-transduced controls. For each independent experiment, knockdown efficiency was calculated relative to the matched scrambled-shRNA control as follows: Knockdown efficiency (%) = [1 − (normalized MT-CO2/GAPDH ratio in the shRNA condition/normalized MT-CO2/GAPDH ratio in the matched scrambled-control condition)] × 100 [57]. Experiment-level knockdown efficiencies were summarized as mean ± SEM across independent experiments. Following confirmation of MT-CO2 suppression, stable knockdown and control cells were used for subsequent PBM treatment and mitochondrial bioenergetic analyses.

### 2.6. Complex IV Activity Assay

Complex IV-dependent respiration was measured in selectively permeabilized BE(2)-C cells using a Agilent Seahorse XF24 Extracellular Flux Analyzer (Agilent Technologies, Santa Clara, CA, USA), as previously described by Sabbir et al. [51] and others [58]. Briefly, control and MT-CO2 knockdown BE(2)-C cells were seeded into Extracellular Flux Analyzer cell culture microplates and allowed to reach approximately 60% confluence. Cells were then exposed to 808 nm NIR light for 10 min every 4 h over a total treatment period of 48 h. At the completion of the light-treatment regimen, cells had reached near-confluence and were subjected to bioenergetic analysis.

At the start of the assay, cells were selectively permeabilized with 10 μM digitonin, allowing exogenous substrates and inhibitors to access the mitochondria while preserving mitochondrial membrane integrity [51]. Uncoupled respiration supported by Complex I was initiated by the addition of 10 mM pyruvate, 2 mM malate, and 4 μM FCCP, resulting in maximal electron transport through Complexes I-IV. Subsequently, 2 μM rotenone and 4 μM antimycin A were added to inhibit Complex I and Complex III, respectively, thereby eliminating electron flow from upstream respiratory complexes. Complex IV-dependent respiration was then stimulated by the addition of 10 mM ascorbate and 100 μM N,N,N′,N′-tetramethyl-p-phenylenediamine (TMPD), which donate electrons directly to cytochrome *c* and drive respiration specifically through Complex IV. Finally, 50 mM sodium azide was added to inhibit Complex IV [59] and confirm the specificity of the measured oxygen consumption rate (OCR) for cytochrome *c* oxidase activity.

Following completion of the assay, the cell culture plates were briefly centrifuged at 300× *g* for 5 min. Cells were then detached by trypsinization, resuspended in 300 μL of basal culture medium, and counted using a hemocytometer and light microscope. OCR values were normalized to 2 × 10^4^ cells based on the measured cell count from each well and expressed as pmol O_2_/min/2 × 10^4^ cells. The OCR remaining after sodium azide treatment was considered residual non-mitochondrial/background oxygen consumption and was subtracted from the respiratory measurements used for quantitative analysis. FCCP induced basal uncoupled (FCCP-stimulated) Complex I–IV-linked respiration was calculated as follows: Basal uncoupled (FCCP-stimulated) Complex I–IV-linked OCR = OCR_pyruvate/malate/FCCP_ − OCR_sodium azide_. Complex IV-dependent respiration was calculated as follows: Complex IV-dependent OCR = OCR _ascorbate/TMPD_ − OCR _sodium azide_, and the resulting values were normalized to the measured post-assay cell number and expressed as pmol O_2_/min/2 × 10^4^ cells.

### 2.7. Statistical Analysis

Statistical analyses were performed using GraphPad Prism version 11.0.2 (GraphPad Software, San Diego, CA, USA). For human brain tissue studies, protein abundance values were normalized to the designated reference sample (A21-006) and analyzed as individual biological observations. Differences in MT-CO2 and SDHB protein abundance among the four diagnostic groups (CN, AD, LBD/PD, and PSP) were evaluated using one-way ANOVA followed by Tukey’s multiple-comparisons test for all pairwise group comparisons. All six pairwise comparisons were included in a single family, with the family-wise significance level set at 0.05. Tukey-adjusted *p*-values and 95% confidence intervals are reported. The homogeneity-of-variance assumption was evaluated using the Brown–Forsythe test. Statistical significance was defined as an adjusted *p* value < 0.05. To compare the diagnostic-group patterns of MT-CO2 and SDHB, the normalized abundance values were additionally analyzed using two-way repeated-measures ANOVA, with protein and diagnosis as factors, including their interaction. Protein abundance was treated as the repeated measure because MT-CO2 and SDHB were quantified in the same subjects. Matching was applied across the two proteins within each subject. The protein × diagnosis interaction was used to determine whether the diagnostic-group pattern differed between MT-CO2 and SDHB. Šídák’s multiple-comparisons test was used to evaluate pairwise diagnostic-group comparisons within each protein, with the 12 comparisons treated as a single family. Within the AD and LBD/PD cohorts, sex-associated differences in MT-CO2 and SDHB abundance were evaluated using unpaired Student’s *t*-tests. Associations of MT-CO2 and SDHB abundance with age at death and postmortem interval were evaluated separately within each cohort using simple linear regression. The corresponding *p* values test whether the regression slopes differ significantly from zero.

For cell-culture experiments, technical replicate wells or immunoblot lanes obtained within the same experimental run were not considered independent biological observations. Technical replicate values were first averaged within each treatment condition and independent experiment, and the resulting experiment-level means were used as the statistical units for inference. For the Seahorse respiration analyses, background-corrected OCR values, obtained after subtraction of the residual non-mitochondrial/background OCR measured after sodium azide, were normalized to the measured post-assay cell number (2 × 10^4^ cells) before averaging within each independent experiment. Each data point in the revised quantitative plots therefore represents one independent experiment. The numbers of independent experiments and technical replicates are specified in the corresponding figure legends.

Comparisons involving multiple groups were analyzed using one-way ANOVA followed by Dunnett’s/Šídák’s multiple-comparisons test. FCCP-stimulated uncoupled Complex I–IV-linked respiration and Complex IV-dependent respiration were analyzed separately using two-way repeated-measures ANOVA, with light treatment and MT-CO2 knockdown status as factors, including their interaction. All four conditions were assayed on the same day within each independent experimental run. Technical-replicate wells were averaged within each condition and experimental run before analysis, and matching was applied across light-treatment conditions within each knockdown status. Šídák’s multiple-comparisons test was used to compare the no-light and 808-nm NIR conditions within scrambled-shRNA and MT-CO2-knockdown cells, with the two comparisons treated as a single family. Technical replicate wells were averaged within each independent experiment before statistical testing. Significant interaction effects were interpreted as evidence that the response to NIR treatment differed according to MT-CO2 expression status. Data are presented as mean ± SEM unless otherwise indicated. Statistical significance was defined as *p* < 0.05.

## 3. Results

### 3.1. MT-CO2 Protein Abundance Is Reduced in the Temporal Cortex of Alzheimer’s Disease Subjects

Gene expression data archived in the Genotype-Tissue Expression (GTEx) database and the Human Protein Atlas indicate that MT-CO2 is highly expressed in normal human brain tissues, exhibiting a ubiquitous cytoplasmic and granular staining pattern. The calculated molecular weight of the human MT-CO2 protein (UniProt: P00403; COX2_HUMAN) is approximately 25.5 kDa. Immunoblot analysis detected MT-CO2-immunoreactive bands at approximately 20 kDa in temporal cortical tissues obtained from individuals with AD, LBD/PD, PSP, and CN control subjects (Figure 1A–D and Figure 2A,B). The difference between the calculated and apparent molecular masses may reflect limited resolution within this molecular-weight range and/or differences in electrophoretic migration associated with the physicochemical properties of the protein. Notably, several previous studies have detected MT-CO2 as an immunoreactive band migrating at approximately 19–20 kDa [60,61,62,63], which is consistent with our observation.

As described in the Methods, the present analysis focused on the MT-CO2 signal generated using the human OXPHOS antibody cocktail. Quantitative analysis revealed a significant overall difference in MT-CO2 protein abundance among the four diagnostic groups (Figure 2C; ordinary one-way ANOVA, $F\left(3,128\right)=6.997$, p=0.0002, $R^{2}=0.1409$). The Brown–Forsythe test did not detect significant heterogeneity of variance among the groups, $F\left(3,128\right)=0.7147$, p=0.5450. Tukey’s multiple-comparisons test showed that MT-CO2 abundance was significantly lower in the AD group than in the CN group (adjusted p=0.0007) and significantly lower in the AD group than in the LBD/PD group (adjusted p=0.0163). The comparisons of CN with LBD/PD (adjusted p=0.1470), CN with PSP (adjusted p=0.1273), AD with PSP (adjusted p=0.5992), and LBD/PD with PSP (adjusted p=0.9376) were not statistically significant. These nonsignificant comparisons do not demonstrate equivalence or the absence of MT-CO2 alterations. Therefore, the present findings demonstrate lower MT-CO2 abundance in AD relative to CN and LBD/PD, whereas the differences involving PSP and the comparisons of LBD/PD or PSP with CN remain uncertain.

To determine whether sex influenced MT-CO2 abundance, cortical protein levels were compared between female (*n* = 38) and male (*n* = 36) subjects with AD and between female (*n* = 8) and male (*n* = 30) subjects with LBD/PD (Figure 3A,B). No statistically significant sex-associated difference in MT-CO2 abundance was detected in either the AD cohort (*p* = 0.1498) or the LBD/PD cohort (*p* = 0.1406; Figure 3A,B). However, the LBD/PD sex comparison should be interpreted cautiously because the female subgroup was small and included two of the three highest values in that cohort. We next examined whether MT-CO2 abundance was associated with age at death or PMI. No significant relationship between age and MT-CO2 abundance was detected in the AD cohort (slope = −0.1764, *p* = 0.7060, *R*^2^ = 0.001989; Figure 3C). In contrast, MT-CO2 abundance showed a significant positive association with age in the LBD/PD cohort (slope = 3.617, *p* = 0.0009, *R*^2^ = 0.2664; Figure 3D). However, because this cohort contained three high observations, the result should be interpreted in light of the cohort size and data distribution. No significant association was detected between MT-CO2 abundance and PMI in either the AD cohort (slope = −0.2476, *p* = 0.6750, *R*^2^ = 0.002456) or the LBD/PD cohort (slope = −2.524, *p* = 0.1766, *R*^2^ = 0.05013; Figure 3E,F). These findings provide no evidence in the present dataset that sex, age at death, or PMI accounted for variation in MT-CO2 abundance within the AD cohort.

To determine whether the AD-associated reduction was restricted to MT-CO2 or accompanied by a change in another OXPHOS protein detected by the same antibody cocktail, we next quantified SDHB abundance across the four diagnostic groups.

### 3.2. SDHB Abundance Is Altered Across Diagnostic Groups, but Its Diagnostic Pattern Differs from That of MT-CO2

The SDHB antibody contained in the OXPHOS cocktail detected an immunoreactive band at approximately 29 kDa, consistent with the expected molecular mass of human SDHB (Figure 4A). Quantitative analysis revealed significant differences in SDHB abundance among the four diagnostic groups (Figure 4B). Tukey’s multiple-comparisons test showed that SDHB abundance was significantly lower in the AD group than in the CN group (adjusted *p* < 0.0001), LBD/PD group (adjusted *p* < 0.0020), and PSP group (adjusted *p* < 0.0026). The comparisons of CN with LBD/PD (adjusted *p* = 0.1209), CN with PSP (adjusted *p* = 0.4834), and LBD/PD with PSP (adjusted *p* = 0.1620) were not statistically significant. Complete pairwise-comparison statistics, including mean differences and 95% confidence intervals, are provided in Supplementary Table S2.

To determine whether MT-CO2 and SDHB exhibited comparable diagnostic-group patterns, their normalized abundance values were jointly analyzed using two-way repeated-measures ANOVA, with the two protein measurements matched within subjects. A significant protein × diagnosis interaction was detected, *F*(3,128) = 6.514, *p* = 0.0004, indicating that the diagnostic-group pattern differed between MT-CO2 and SDHB. Significant main effects of protein, *F*(1,128) = 80.69, *p* < 0.0001, and diagnosis, *F*(3,128) = 9.065, *p* < 0.0001, were also observed (Supplementary Table S3).

Šídák-adjusted comparisons within each protein showed that SDHB abundance was significantly lower in AD than in CN (adjusted *p* = 0.0001) and PSP (adjusted *p* = 0.0228). SDHB abundance was also significantly lower in LBD/PD than in CN (adjusted *p* = 0.0185). The differences between AD and LBD/PD (adjusted *p* = 0.4777), CN and PSP (adjusted *p* = 0.9436), and LBD/PD and PSP (adjusted *p* = 0.5772) were not statistically significant (Supplementary Table S3).

Within the same analysis, MT-CO2 abundance was significantly lower in AD than in CN (Šídák-adjusted *p* = 0.0002) and LBD/PD (adjusted *p* = 0.0110). The comparisons of CN with LBD/PD (adjusted *p* = 0.1976), CN with PSP (adjusted *p* = 0.1641), AD with PSP (adjusted *p* = 0.8885), and LBD/PD with PSP (adjusted *p* = 0.9998) were not statistically significant (Supplementary Table S3).

Thus, SDHB was also reduced in AD relative to CN, indicating that the AD-associated OXPHOS alteration was not restricted to MT-CO2 among the two proteins quantified. However, the significant protein × diagnosis interaction and the differing pairwise-comparison patterns demonstrate that MT-CO2 and SDHB were not affected identically across the diagnostic groups. Because mitochondrial content was not independently measured, these findings do not establish that the observed protein alterations reflect a generalized reduction in mitochondrial abundance.

We next examined whether SDHB abundance was associated with sex, age at death, or PMI (Figure 5). No statistically significant sex-associated difference in SDHB abundance was detected in either the AD cohort (*p* = 0.5174) or the LBD/PD cohort (*p* = 0.0804; Figure 5A,B). SDHB abundance showed a significant negative association with age at death in the AD cohort (slope = −1.039, *p* = 0.0033; Figure 5C) and a significant positive association with age at death in the LBD/PD cohort (slope = 1.855, *p* = 0.0198; Figure 5D). No significant association was detected between SDHB abundance and PMI in either the AD cohort (slope = −1.1993, *p* = 0.6643) or the LBD/PD cohort (slope = −1.950, *p* = 0.1366; Figure 5E,F). These findings indicate that SDHB abundance was associated with age, but not sex or PMI, within the AD cohort. However, the within-cohort association with age does not establish that age accounts for the difference in SDHB abundance between AD and CN.

### 3.3. Red and Near-Infrared Light Treatment Induces an Exposure-Duration-Dependent Increase in MT-CO2 Expression in BE(2)-C Cells

Previous microarray-based gene expression analyses of human fibroblasts irradiated with 628 nm red light have demonstrated the upregulation of multiple genes involved in mitochondrial bioenergetics, including components of the electron transport chain [64]. Based on these observations, we sought to determine whether PBM could similarly enhance MT-CO2 expression in human neuronal cells. To investigate this possibility, BE(2)-C human neuroblastoma cells were exposed to 650 nm red light or 808 nm near-infrared light for varying durations (2–30 min). Immunoblot analysis demonstrated that MT-CO2 protein abundance varied with exposure duration following repeated treatment with either wavelength (Figure 6A–C). For 650-nm red-light treatment, the 2-min exposure did not significantly alter MT-CO2 abundance relative to no light, whereas the 5-, 10-, 15-, and 20-min exposures produced significant increases; the increase following the 30-min exposure was not statistically significant (Figure 6B). For 808-nm NIR-light treatment, the 2-min exposure was not significantly different from no light, whereas the 5-, 10-, 15-, 20-, and 30-min exposures significantly increased MT-CO2 abundance (Figure 6C). The largest responses to both wavelengths were observed within the 10–15-min exposure range. Together, these findings demonstrate wavelength-associated, non-monotonic exposure-duration responses, with the NIR-induced increase remaining significant at 30 min.

To directly compare the effects of red and NIR light under the same exposure condition, MT-CO2 abundance was evaluated in matched experiments following repeated 10-min treatments with 650-nm red light or 808-nm NIR light administered every 4 h over 48 h (Figure 6D). The mean normalized MT-CO2/ATP5A ratio increased from 0.565 ± 0.072 in the no-light condition to 0.917 ± 0.101 following red-light treatment and 1.177 ± 0.149 following NIR-light treatment (mean ± SEM; *n* = 3 independent experiments). Analysis of the log-transformed ratios using repeated-measures one-way ANOVA followed by Tukey’s multiple-comparisons test showed significantly increased MT-CO2 abundance following both red-light and NIR-light treatment compared with the no-light condition (adjusted *p* = 0.0013 and adjusted *p* < 0.0001, respectively). MT-CO2 abundance was also significantly greater following NIR-light treatment than following red-light treatment (adjusted *p* = 0.0098). Thus, under the repeated 10-min treatment condition, both wavelengths increased MT-CO2 abundance, with 808-nm NIR light producing a greater increase than 650-nm red light.

### 3.4. Knockdown of MT-CO2 Significantly Reduced NIR-Induced Complex IV Respiration in BE(2)-C Cells

Previous studies have demonstrated that 808 nm NIR light, which is absorbed by ETC Complex IV, can acutely enhance Complex IV-dependent oxygen consumption and ATP production [65]. Therefore, to determine whether the light-induced upregulation of MT-CO2 contributes functionally to enhanced Complex IV activity, we first generated MT-CO2 knockdown BE(2)-C cells using shRNA-mediated gene silencing. Two previously validated MT-CO2-targeting shRNA constructs were evaluated (Figure 7A,B) [55].

Compared with the matched scrambled shRNA-transduced control within each independent experiment, MT-CO2 protein abundance was reduced to 28.8 ± 0.6% and 52.7 ± 3.0% (mean ± SEM) by shRNA Sequence 1 and Sequence 2, respectively, corresponding to knockdown efficiencies of approximately 71% and 47% (Figure 7B). Because shRNA sequence 1 produced the most robust suppression of MT-CO2 expression, it was selected for all subsequent experiments. Furthermore, although both 650 nm red light and 808 nm NIR light significantly increased MT-CO2 protein abundance, subsequent bioenergetic analyses were performed using only the 808 nm treatment because of its well-established ability to directly enhance Complex IV-dependent mitochondrial respiration and ATP production [65].

To evaluate the functional contribution of MT-CO2 to Complex IV activity, we performed a Complex IV respiration assay in digitonin-permeabilized BE(2)-C cells using a previously validated protocol [51]. Selective permeabilization of the plasma membrane allows respiratory substrates and inhibitors direct access to the mitochondria while preserving mitochondrial integrity [51]. As expected, the addition of pyruvate, malate, and FCCP stimulated robust uncoupled respiration driven by Complexes I-IV in scrambled shRNA control cells (Figure 7C,D). In contrast, basal uncoupled (FCCP-stimulated) Complex I-IV-linked respiration was markedly reduced in MT-CO2 knockdown cells, indicating impaired mitochondrial respiratory capacity (Figure 7C,D). Treatment with 808 nm NIR light significantly increased basal uncoupled (FCCP-stimulated) respiration in scrambled shRNA control cells; however, this stimulatory effect was substantially attenuated in MT-CO2 knockdown cells (Figure 7C,D). Subsequent addition of rotenone and antimycin A inhibited Complex I- and Complex III-mediated electron transport (Figure 7C), thereby eliminating respiration originating from upstream ETC complexes. The addition of ascorbate and TMPD then restored electron flow directly to cytochrome *c*, enabling selective measurement of Complex IV-dependent oxygen consumption. Notably, MT-CO2 knockdown significantly reduced ascorbate/TMPD-stimulated respiration compared with scrambled shRNA controls (Figure 7E), supporting a contribution of MT-CO2 to the maintenance of Complex IV activity. These findings indicate that reduced MT-CO2 abundance is associated with impaired basal Complex IV activity and an attenuated bioenergetic response to NIR PBM, supporting a contribution of MT-CO2 to both processes.

Two-way repeated-measures ANOVA revealed significant interactions between light treatment and MT-CO2 knockdown status for both FCCP-stimulated uncoupled Complex I–IV-linked respiration, *F*(1, 6) = 11.97, *p* = 0.0135 (Figure 7D, Supplementary Table S5), and Complex IV-dependent respiration, *F*(1, 6) = 98.83, *p* < 0.0001 (Figure 7E, Supplementary Table S5). Šídák-adjusted comparisons showed that NIR significantly increased FCCP-stimulated uncoupled Complex I–IV-linked respiration and Complex IV-dependent respiration in scrambled-shRNA cells (adjusted *p* = 0.0014 and *p* < 0.0001, respectively). NIR did not significantly alter basal uncoupled (FCCP-stimulated) Complex I–IV-linked respiration in MT-CO2-knockdown cells (adjusted *p* = 0.9664). Although NIR produced an approximately 22% numerical increase in Complex IV-dependent respiration in knockdown cells, this comparison was not statistically significant (adjusted *p* = 0.6688). Thus, MT-CO2 knockdown markedly attenuated the NIR-induced respiratory response.

### 3.5. Effects of Photobiomodulation on MT-CO1, MT-CO3, and CYCS Abundance

Mammalian Complex IV is composed of 14 subunits, including the three mitochondrially encoded catalytic core proteins MT-CO1, MT-CO2, and MT-CO3 and eleven nuclear-encoded subunits [66]. Because MT-CO2 abundance increased significantly following red- and NIR-light treatment, we investigated whether this response was accompanied by changes in the other mitochondrially encoded catalytic core subunits, MT-CO1 and MT-CO3 (Figure 8A). We also examined CYCS, the mobile electron carrier that transfers electrons from Complex III to Complex IV.

Although the calculated molecular mass of MT-CO1 is approximately 57 kDa, the antibody used in this study (Cell Signaling Technology, catalog no. 55159) has a supplier-reported endogenous molecular weight of approximately 32 kDa. Previous studies have also reported apparent MT-CO1 migration at approximately 39.5–40 kDa under their respective SDS-PAGE conditions [67,68]. The MT-CO1 band quantified in the present study migrated at approximately 37 kDa (Figure 8B), consistent with the supplier-reported value and with previously documented lower-than-calculated apparent molecular weights for MT-CO1. The calculated molecular masses of MT-CO3 and CYCS are approximately 29.9 and 11.7 kDa, respectively. The supplier reports a theoretical molecular weight of approximately 30 kDa for MT-CO3 and an endogenous molecular weight of approximately 14 kDa for CYCS. The corresponding immunoreactive bands migrated at approximately 25–30 and 14–15 kDa, respectively, under the electrophoretic conditions used in this study (Figure 8C,D). Quantitative analysis revealed that neither 650 nm red light nor 808 nm NIR light significantly altered MT-CO1, MT-CO3, or CYCS abundance compared with the matched no-light controls (Figure 8F–H). In contrast to the increase observed for MT-CO2, the abundance of MT-CO1, MT-CO3, and CYCS remained unchanged under the treatment conditions examined.

Collectively, these findings indicate that the PBM-induced increase in MT-CO2 was not accompanied by corresponding changes in the other mitochondrially encoded catalytic core subunits, MT-CO1 and MT-CO3, or in CYCS abundance. Under the conditions examined, the protein-abundance response therefore appeared selective for MT-CO2 among the mitochondrial proteins evaluated.

## 4. Discussion

Mitochondrial dysfunction has long been recognized as a hallmark of AD [17,69], yet the specific mitochondrial proteins contributing to disease progression remain incompletely understood. In the present study, MT-CO2 protein abundance was significantly lower in the temporal cortex of AD subjects than in CN and LBD/PD subjects. SDHB abundance was also lower in AD than in CN, indicating that the AD-associated OXPHOS alteration was not restricted to MT-CO2 among the two proteins quantified. However, the significant protein × diagnosis interaction demonstrated that MT-CO2 and SDHB exhibited distinct diagnostic-group patterns. We further demonstrated that PBM using red and NIR light increases MT-CO2 abundance in neuroblastoma cells. Functional studies indicated that MT-CO2 contributes to Complex IV-dependent respiration and to the bioenergetic response induced by 808 nm NIR light. Collectively, these findings identify reduced MT-CO2 and SDHB abundance as a feature of AD temporal cortex and demonstrate that PBM can modulate MT-CO2 abundance and mitochondrial respiration in a neuroblastoma cell model. Nevertheless, the human cohort comparisons do not establish that either protein alteration is specific to AD, nor do they establish that the observed changes reflect a generalized loss of mitochondrial content. A direct mechanistic link between PBM and restoration of MT-CO2 or other mitochondrial protein deficiency in AD remains to be established.

The observation that MT-CO2 protein abundance is reduced in the AD temporal cortex is consistent with the mitochondrial cascade hypothesis, which proposes that mitochondrial dysfunction is an early pathogenic event contributing to neurodegeneration and disease progression [17]. Numerous studies have reported abnormalities in mitochondrial respiration and Complex IV function in AD. Reduced expression of oxidative phosphorylation-related transcripts, including mitochondrial respiratory genes, has been documented in the AD brain, while diminished Complex IV activity has been reported in platelets from AD patients and individuals with mild cognitive impairment [28,70,71,72]. Transgenic mouse models of AD exhibit progressive deficits in Complex IV activity. Neuronal overexpression of mutant amyloid precursor protein (APP) reduces cytochrome *c* oxidase activity [73], and studies using APP/PS2 and triple-transgenic AD mice have shown age-dependent declines in the Complex IV/citrate synthase activity ratio, which become more pronounced as pathology progresses [74]. Proteomic analysis of the same triple-transgenic AD model identified MT-CO2 as one of the differentially expressed mitochondrial proteins relative to wild-type controls [74], providing experimental support for our observation of reduced MT-CO2 abundance in postmortem AD brain tissue. Additional evidence linking Complex IV dysfunction to AD pathology comes from studies demonstrating that Aβ42 oligomers can directly inhibit cytochrome *c* oxidase activity in a copper-dependent manner [75].

MT-CO2 forms part of the mitochondrially encoded catalytic core of cytochrome *c* oxidase and participates in electron transfer from cytochrome *c* toward the catalytic center of Complex IV [66]. Reduced MT-CO2 abundance could therefore compromise electron-transfer efficiency, ATP production, and overall respiratory-chain function. The resulting bioenergetic impairment could contribute to oxidative stress, synaptic dysfunction, and neuronal loss, all of which are prominent pathological features of AD. The present findings extend previous evidence of Complex IV dysfunction in AD by identifying MT-CO2 as a catalytic subunit with reduced abundance in AD temporal cortex. However, the postmortem analysis is associative and does not establish whether reduced MT-CO2 is a cause, consequence, or accompanying feature of AD-associated mitochondrial dysfunction.

The additional SDHB analysis provides evidence that the AD-associated OXPHOS alteration was not restricted to MT-CO2 among the two proteins quantified. SDHB is a nuclear-encoded component of respiratory-chain Complex II, whereas MT-CO2 is a mitochondrially encoded catalytic subunit of Complex IV. The finding that both proteins were reduced in AD relative to CN therefore suggests that the postmortem alteration may involve more than a single Complex IV subunit. Nevertheless, the significant protein × diagnosis interaction indicates that MT-CO2 and SDHB did not exhibit uniform changes across the diagnostic groups. In the joint analysis, SDHB abundance was lower in AD than in CN and PSP and lower in LBD/PD than in CN, whereas MT-CO2 abundance was lower in AD than in CN and LBD/PD. These findings argue against interpreting the results as an equivalent reduction in MT-CO2 and SDHB across neurodegenerative disorders. Instead, the data suggest that the abundance of individual OXPHOS proteins may be affected differently according to disease context.

Within the AD cohort, MT-CO2 abundance was not significantly associated with age, sex, or PMI. These findings indicate that the measured demographic and postmortem variables did not explain the variation in MT-CO2 abundance within the AD group. SDHB abundance was likewise not significantly associated with sex or PMI in the AD cohort; however, it showed a significant negative association with age at death. Thus, unlike MT-CO2, variation in SDHB abundance within the AD cohort was associated with age. In the LBD/PD cohort, both MT-CO2 and SDHB showed significant positive associations with age. These relationships should be interpreted cautiously in view of the cohort sizes and data distributions. Moreover, because the regression analyses were performed within individual diagnostic groups, they do not determine whether age accounts for the differences in protein abundance among those groups. The limited number of CN subjects also precluded a robust age-stratified analysis in cognitively normal individuals.

The disease-group comparisons likewise do not establish disease specificity. MT-CO2 abundance was lower in AD than in CN and LBD/PD, but AD did not differ significantly from PSP, and neither LBD/PD nor PSP differed significantly from CN in the primary one-way analysis. SDHB was reduced in AD relative to CN, but its pattern across the other diagnostic groups differed from that of MT-CO2. Nonsignificant comparisons do not establish equivalence or the absence of protein alterations. Accordingly, the present findings identify changes in MT-CO2 and SDHB abundance in AD temporal cortex but do not demonstrate that these changes are unique to AD or absent from other neurodegenerative disorders.

A major finding of the present study is that both 650 nm red light and 808 nm NIR light significantly increased MT-CO2 protein abundance in BE(2)-C neuroblastoma cells. PBM is widely believed to act through cytochrome c oxidase [76], yet whether PBM alters the expression of individual Complex IV subunits has remained unclear. Our data demonstrate that PBM does not simply enhance mitochondrial function acutely but can also induce changes in the abundance of a specific mitochondrially encoded catalytic component of Complex IV. This finding provides new insight into how repeated light exposure may produce sustained bioenergetic benefits beyond the immediate photochemical effects traditionally attributed to cytochrome c oxidase activation.

The functional importance of MT-CO2 was supported through shRNA-mediated knockdown experiments. Reduction in MT-CO2 protein expression resulted in a substantial decrease in Complex IV-dependent oxygen consumption, demonstrating that MT-CO2 contributes to normal respiratory-chain function. These findings are consistent with previous reports showing that pathogenic MT-CO2 mutations cause mitochondrial disorders characterized by neurological dysfunction and impaired Complex IV activity [30,31]. Because neurons depend heavily on oxidative phosphorylation for ATP generation, disruption of Complex IV function could have substantial consequences for neuronal homeostasis and survival. The knockdown findings therefore provide a mechanistic basis for the hypothesis that reduced MT-CO2 abundance may contribute to mitochondrial dysfunction in AD, although this relationship was not tested directly in AD-derived cells or tissues.

Notably, the increase in Complex IV-dependent respiration induced by 808 nm NIR light in scrambled-shRNA cells was markedly attenuated following MT-CO2 knockdown. The significant interaction between light treatment and MT-CO2 knockdown status indicates that the respiratory response to NIR treatment differed according to MT-CO2 abundance. Although NIR treatment produced an approximately 22% numerical increase in Complex IV-dependent respiration in knockdown cells, this change was not statistically significant. These findings suggest that MT-CO2 contributes to the full bioenergetic response to NIR PBM under the experimental conditions examined. Previous PBM studies have primarily considered cytochrome *c* oxidase as an intact enzyme complex and have emphasized its heme and copper centers as potential photoacceptors [37,65,75]. The present results suggest that the integrity and abundance of individual catalytic subunits, particularly MT-CO2, may also influence PBM-associated mitochondrial responses.

In the cell-culture PBM experiments, the light-induced increase appeared comparatively selective for MT-CO2 among the mitochondrial proteins evaluated. Neither of the other mitochondrially encoded Complex IV catalytic core subunits, MT-CO1 and MT-CO3, showed significant changes following light treatment. CYCS abundance also remained unchanged. These observations argue against a generalized increase in the abundance of the mitochondrial electron-transport proteins examined and instead suggest a comparatively selective effect on MT-CO2. Although the mechanism underlying this specificity remains unclear, it raises the possibility that PBM influences mitochondrial gene expression, transcript stability, protein turnover, or assembly of Complex IV in a subunit-specific manner. Future studies examining mitochondrial transcriptional regulation and Complex IV assembly dynamics following PBM will be necessary to address these possibilities.

Another important implication of the present findings concerns the interpretation of optogenetic studies. Optogenetics combines genetically encoded light-sensitive proteins with wavelength-specific illumination to control cellular activity with high spatial and temporal precision [77]. In neuronal studies, plasma membrane-localized channelrhodopsins are commonly activated by blue light (~470–480 nm) to induce membrane depolarization and neuronal excitation, whereas light-driven inhibitory opsins, including halorhodopsins, can be used to suppress neuronal activity [78]. Red-shifted channelrhodopsins are particularly relevant because their longer activation wavelengths provide greater tissue penetration and can enable combination with blue-light-responsive optogenetic tools [79]. Optogenetic strategies have also been extended to mitochondria, where mitochondria-targeted opsins can modulate mitochondrial membrane potential, oxidative phosphorylation, ATP production, Ca^2+^ signaling, and respiratory metabolism [80]. Importantly, however, illumination used to activate an optogenetic actuator may not necessarily be biologically inert and could potentially influence endogenous cellular photoacceptors independently of the engineered construct. Although optogenetic wavelengths and illumination parameters differ from the PBM conditions used here, our finding that 650-nm red and 808-nm NIR light altered MT-CO2 abundance and mitochondrial respiration in the absence of an optogenetic construct raises the possibility that optogenetic and direct photobiomodulatory effects could coexist under certain illumination conditions. Future studies should therefore investigate this potential interaction and distinguish actuator-dependent responses from direct wavelength- and dose-dependent effects of light using appropriate light-only, construct-only, and wavelength- and dose-matched controls.

Several limitations should be considered when interpreting the present findings. First, the CN cohort was relatively small compared with the AD and LBD/PD cohorts, and the PSP cohort included only 11 subjects. These unequal group sizes reduce the precision of the control and PSP estimates and limit the interpretation of between-disease comparisons. The inclusion of LBD/PD and PSP cohorts permitted exploratory comparisons across neurodegenerative disorders, but these comparisons cannot establish disease specificity. Validation in larger, more balanced, and independent postmortem cohorts will therefore be necessary.

The SDHB analysis partially addressed whether the lower MT-CO2 abundance in AD was accompanied by an alteration in another OXPHOS protein. SDHB was also lower in AD than in CN, indicating that the alteration was not restricted to MT-CO2 among the two proteins quantified. However, SDHB represents a single additional respiratory-chain protein and is not an independent measure of mitochondrial content. Moreover, the significant protein × diagnosis interaction indicates that SDHB and MT-CO2 did not exhibit identical diagnostic-group patterns. Therefore, the present findings cannot distinguish whether the observed changes reflect a broader reduction in mitochondrial abundance, protein-specific dysregulation, or a combination of these processes. Measurements of established mitochondrial-content indices, such as citrate synthase activity, mitochondrial DNA copy number, or additional mitochondrial proteins, will be required to resolve this question.

Although two independent shRNA constructs reduced MT-CO2 abundance, only shRNA sequence 1, which produced the more robust and reproducible knockdown, was used in the functional respiration experiments. Sequence-specific or off-target effects therefore cannot be excluded completely. Replication using the second independent shRNA, together with restoration of MT-CO2 expression in knockdown cells, would provide stronger evidence that the respiratory phenotype and attenuated PBM response resulted specifically from MT-CO2 suppression.

MT-CO2 was assessed primarily at the protein level, and the molecular mechanism underlying its PBM-associated increase was not investigated. It remains unclear whether PBM regulates MT-CO2 through mitochondrial transcription, translation, transcript stability, protein stability, turnover, or assembly into the Complex IV holoenzyme. In addition, although the human-tissue and cell-culture findings address complementary aspects of MT-CO2 biology, the mechanistic connection between them remains indirect. The postmortem analysis identifies alterations in MT-CO2 and SDHB abundance in AD temporal cortex, whereas the BE(2)-C experiments show that PBM increases MT-CO2 abundance and that MT-CO2 contributes to PBM-enhanced mitochondrial respiration under controlled conditions. The present study does not demonstrate that PBM restores MT-CO2 deficiency or mitochondrial function in AD-derived neurons or human AD brain tissue.

Finally, the retrospective and cross-sectional nature of the human-tissue analysis precludes conclusions regarding causality or the temporal relationship between altered protein abundance and disease progression. Studies using AD-relevant cellular systems, patient-derived neurons, and in vivo AD models will be necessary to determine whether PBM can restore AD-associated MT-CO2 deficiency and improve mitochondrial bioenergetic function in a disease-relevant context. Longitudinal studies and analyses of pathologically stratified postmortem cohorts will also be required to determine whether alterations in MT-CO2 and SDHB emerge early in the disease process, vary with neuropathological severity, or develop as consequences of progressive neurodegeneration. Despite these limitations, the present study broadens the postmortem findings beyond MT-CO2 while demonstrating that MT-CO2 and SDHB exhibit distinct diagnostic-group patterns. It further provides functional evidence that MT-CO2 contributes to Complex IV-dependent respiration and the bioenergetic response to NIR PBM.

## 5. Conclusions

In conclusion, this study identifies alterations in MT-CO2 and SDHB abundance in AD temporal cortex and provides evidence that MT-CO2 contributes to Complex IV-dependent respiration and the mitochondrial bioenergetic response to PBM. SDHB was also reduced in AD relative to CN, indicating that the AD-associated OXPHOS alteration was not restricted to MT-CO2 among the two proteins quantified. However, the distinct diagnostic-group patterns of MT-CO2 and SDHB and the absence of an independent measure of mitochondrial content preclude attributing these findings to a generalized loss of mitochondrial abundance. Repeated treatment with 650 nm red light or 808 nm NIR light increased MT-CO2 abundance in BE(2)-C neuroblastoma cells, with the largest responses observed following the repeated 10- and 15-min exposure conditions. The non-monotonic exposure-duration pattern indicates that longer light exposure does not necessarily produce proportionally greater biological responses. shRNA-mediated reduction in MT-CO2 was associated with impaired Complex IV-dependent respiration and attenuation of the NIR-induced respiratory response. Together, these findings support a potential contribution of MT-CO2 to AD-associated mitochondrial dysfunction and demonstrate that PBM can modulate MT-CO2 abundance and mitochondrial bioenergetic function in a neuroblastoma cell model. Further studies using additional gene-silencing constructs, rescue experiments, and AD-relevant experimental systems are needed to establish the specificity and disease relevance of these observations. Given the growing use of optogenetic approaches to modulate neuronal and mitochondrial functions, it will also be important to determine whether optogenetic illumination itself influences endogenous mitochondrial pathways and thereby interacts with photobiomodulatory responses.

## Figures and Tables

**Figure 1:**
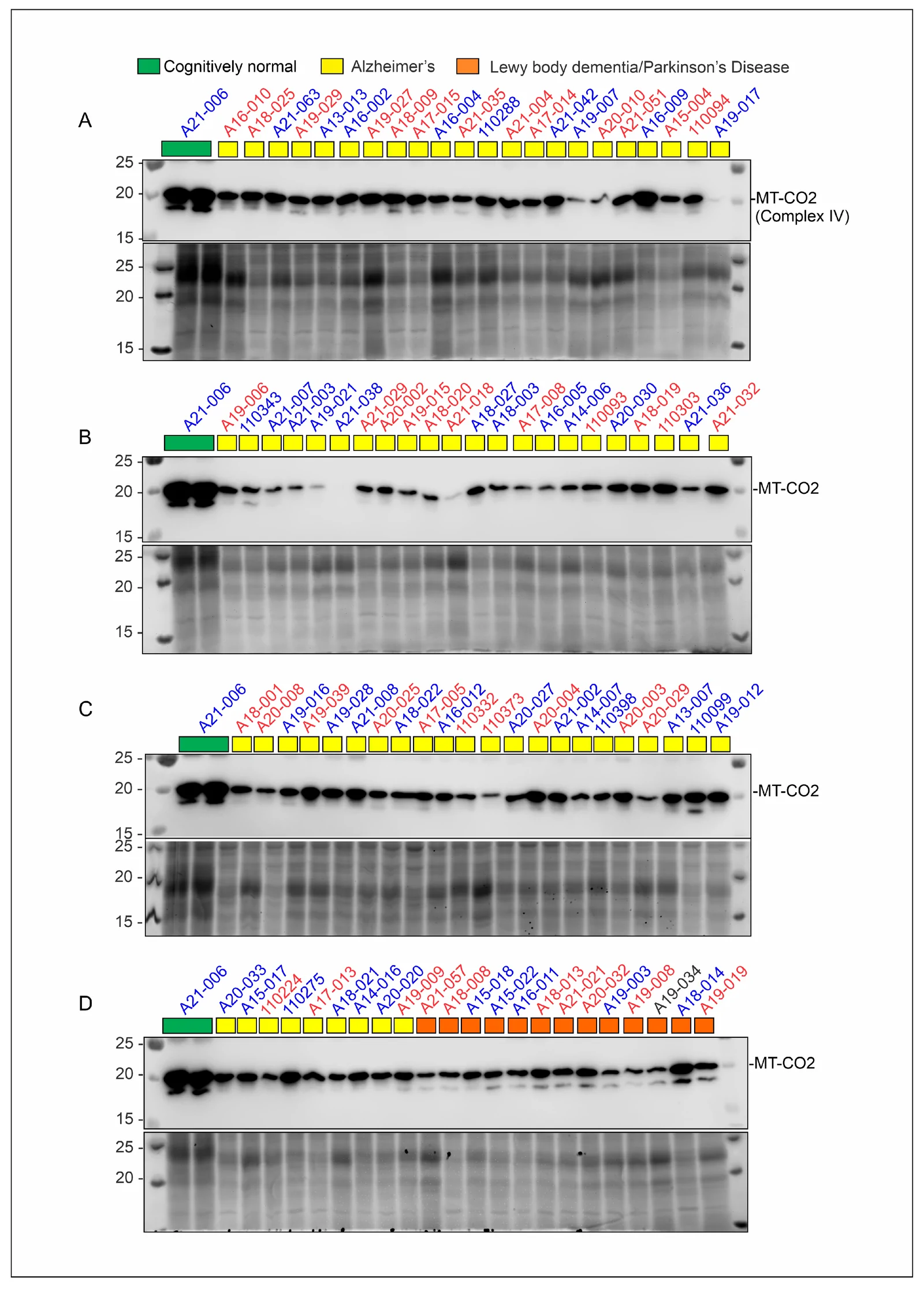
**Representative immunoblot analysis of MT-CO2 protein expression in the human temporal cortex across neurodegenerative disease cohorts**. (**A**–**D**) Representative immunoblots showing the relative abundance of MT-CO2 in postmortem human temporal cortex tissues. Sample identification numbers are indicated above each immunoblot. Female and male subjects are highlighted in blue and red, respectively. Samples from CN individuals, AD patients, and LBD/PD patients are distinguished by different color codes, as indicated in the figure. The corresponding bottom-panel Oriole-stained gels demonstrate equivalent protein loading for the samples shown in the immunoblots above.

**Figure 2:**
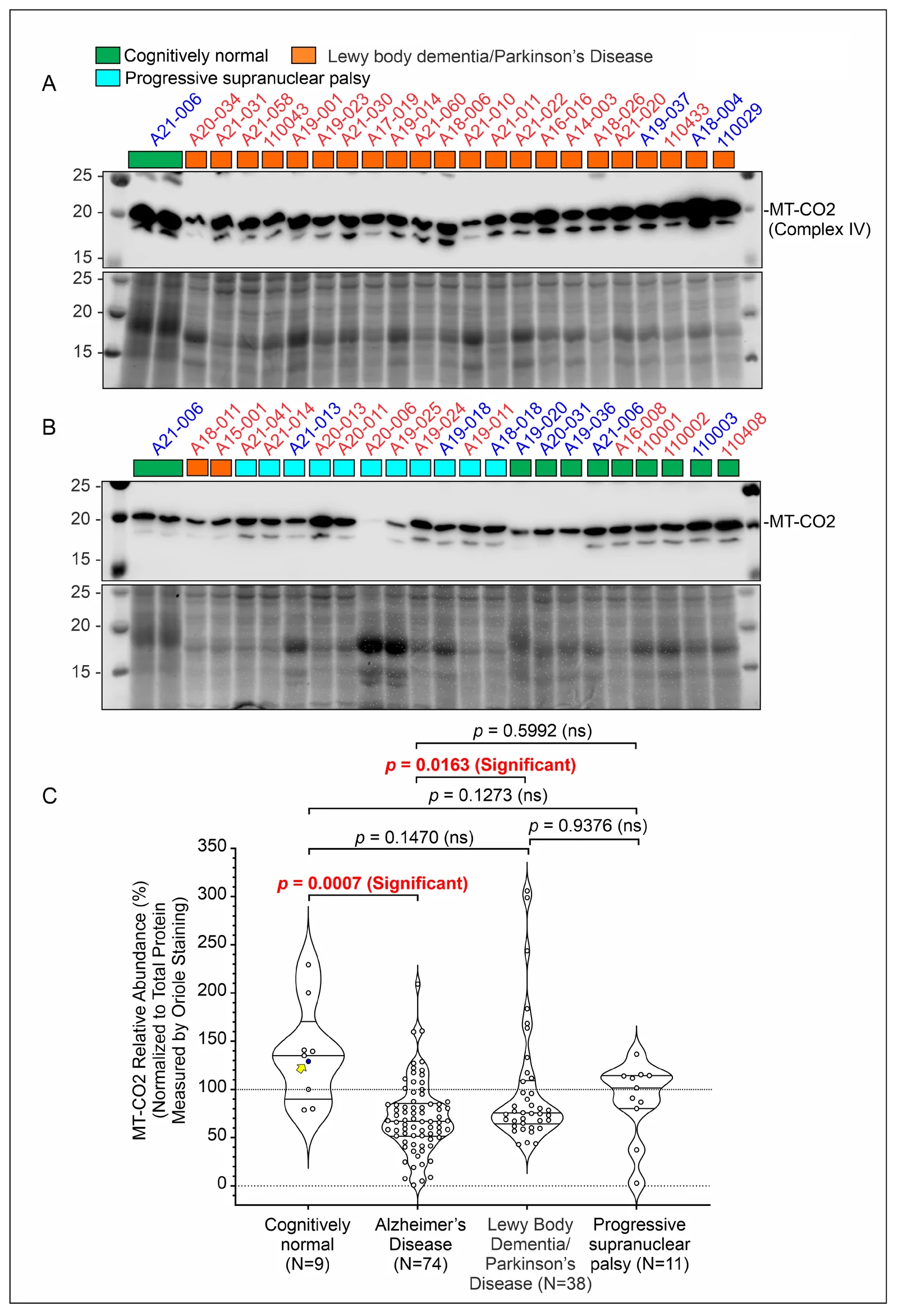
**MT-CO2 protein abundance in the temporal cortex of Alzheimer’s disease and other neurodegenerative disorders.** (**A**,**B**) Representative immunoblots showing MT-CO2 abundance in postmortem temporal cortex tissues. Sample identification numbers are shown above the blots; blue and red indicate female and male subjects, respectively, and colors distinguish the diagnostic groups. Corresponding Oriole-stained gels demonstrate protein loading. (**C**) Violin scatter plot of MT-CO2 abundance in cognitively normal controls (CN; *n* = 9), Alzheimer’s disease (AD; *n* = 74), Lewy body dementia/Parkinson’s disease (LBD/PD; *n* = 38), and progressive supranuclear palsy (PSP; *n* = 11). MT-CO2 abundance was expressed relative to the common inter-blot reference lysate from CN subject A21-006, which was assigned a value of 100%. Each point represents one subject and is the mean of three experimental runs performed using the same tissue lysate, with one or two technical replicate lanes per run. The blue filled point and yellow arrow identify a separate lysate prepared from a different tissue fraction from subject A21-006; this sample had an MT-CO2 abundance of 129.1 ± 1.520% relative to the common reference lysate (mean ± SEM across three experimental runs). Differences among diagnostic groups were analyzed using ordinary one-way ANOVA followed by Tukey’s multiple-comparisons test [*F*(3,128) = 6.997, *p* = 0.0002]. Horizontal bars indicate the significant CN-versus-AD and AD-versus-LBD/PD comparisons. Exact adjusted *p* values and complete pairwise-comparison statistics are provided in Supplementary Table S1.

**Figure 3:**
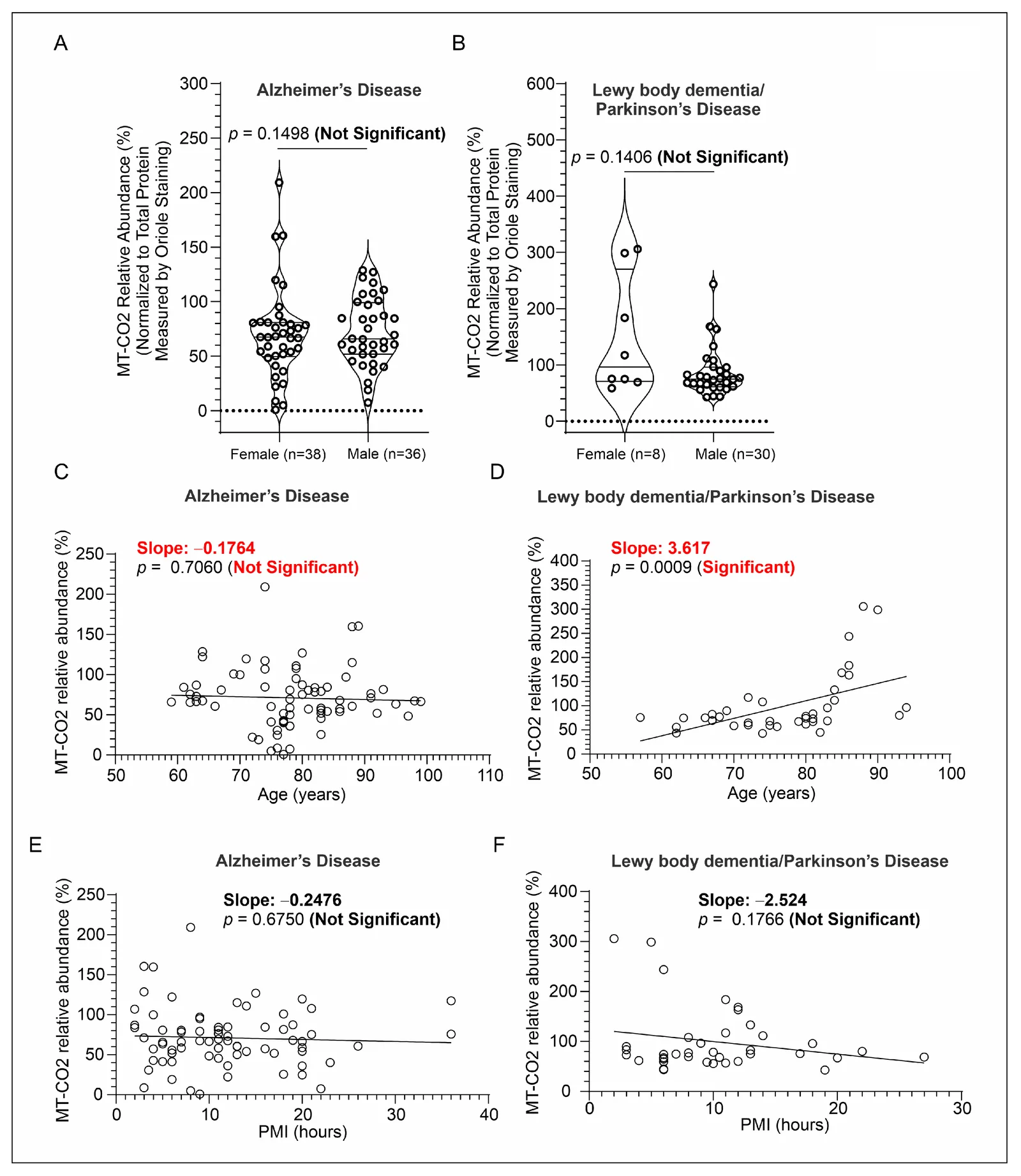
**Associations of MT-CO2 protein abundance with sex, age at death, and postmortem interval in the AD and LBD/PD cohorts.** (**A**,**B**) Violin scatter plots showing temporal cortical MT-CO2 protein abundance in female (*n* = 38) and male (*n* = 36) subjects with AD (**A**) and in female (*n* = 8) and male (*n* = 30) subjects with LBD/PD (**B**). Differences between female and male subjects were analyzed using unpaired Student’s *t*-tests. (**C**,**D**) Scatter plots showing the relationships between age at death and MT-CO2 protein abundance in the AD (**C**) and LBD/PD (**D**) cohorts. (**E**,**F**) Scatter plots showing the relationships between postmortem interval (PMI) and MT-CO2 protein abundance in the AD (**E**) and LBD/PD (**F**) cohorts. Each data point represents one subject. Solid black lines in panels (**C**–**F**) represent simple linear regression fits generated using GraphPad Prism. The reported *p*-values for panels (**C**–**F**) test whether the corresponding regression slopes differ significantly from zero.

**Figure 4:**
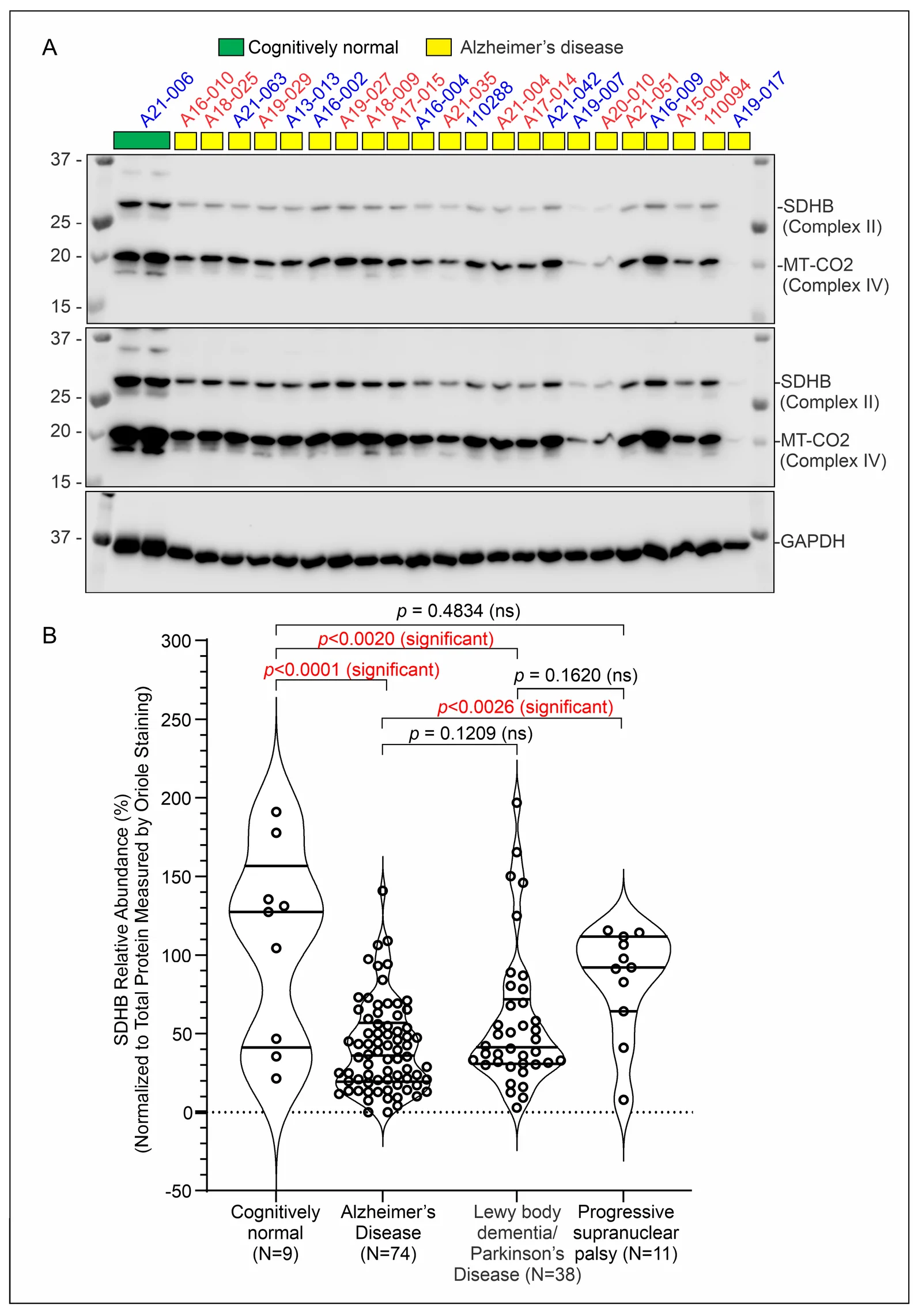
**SDHB protein abundance in the temporal cortex of Alzheimer’s disease and other neurodegenerative disorders.** (**A**) Representative OXPHOS-cocktail immunoblot images showing SDHB and MT-CO2 abundance in postmortem temporal cortex tissues from CN reference subject A21-006 and subjects with AD. SDHB immunoblot images for the remaining subjects are provided in the supplementary raw-image files. Sample identification numbers are shown above the blots; blue and red indicate female and male subjects, respectively, and the colored boxes distinguish the diagnostic groups. Two exposures of the same immunoblot are shown to facilitate visualization of the SDHB and MT-CO2 signals. The corresponding GAPDH immunoblot is shown below as an additional reference but was not used for SDHB normalization. (**B**) Violin scatter plot showing SDHB abundance in CN (*n* = 9), AD (*n* = 74), LBD/PD (*n* = 38), and PSP (*n* = 11). Background-corrected SDHB band intensity was normalized to the corresponding Oriole-stained total-protein intensity and expressed relative to the common inter-blot reference lysate from CN subject A21-006, which was assigned a value of 100%. Each point represents one subject and is the mean of three experimental runs performed using the same tissue lysate, with one or two technical-replicate lanes per run. Differences among diagnostic groups were analyzed using ordinary one-way ANOVA followed by Tukey’s multiple-comparisons test for all pairwise comparisons. Exact Tukey-adjusted *p* values are shown in the figure.

**Figure 5:**
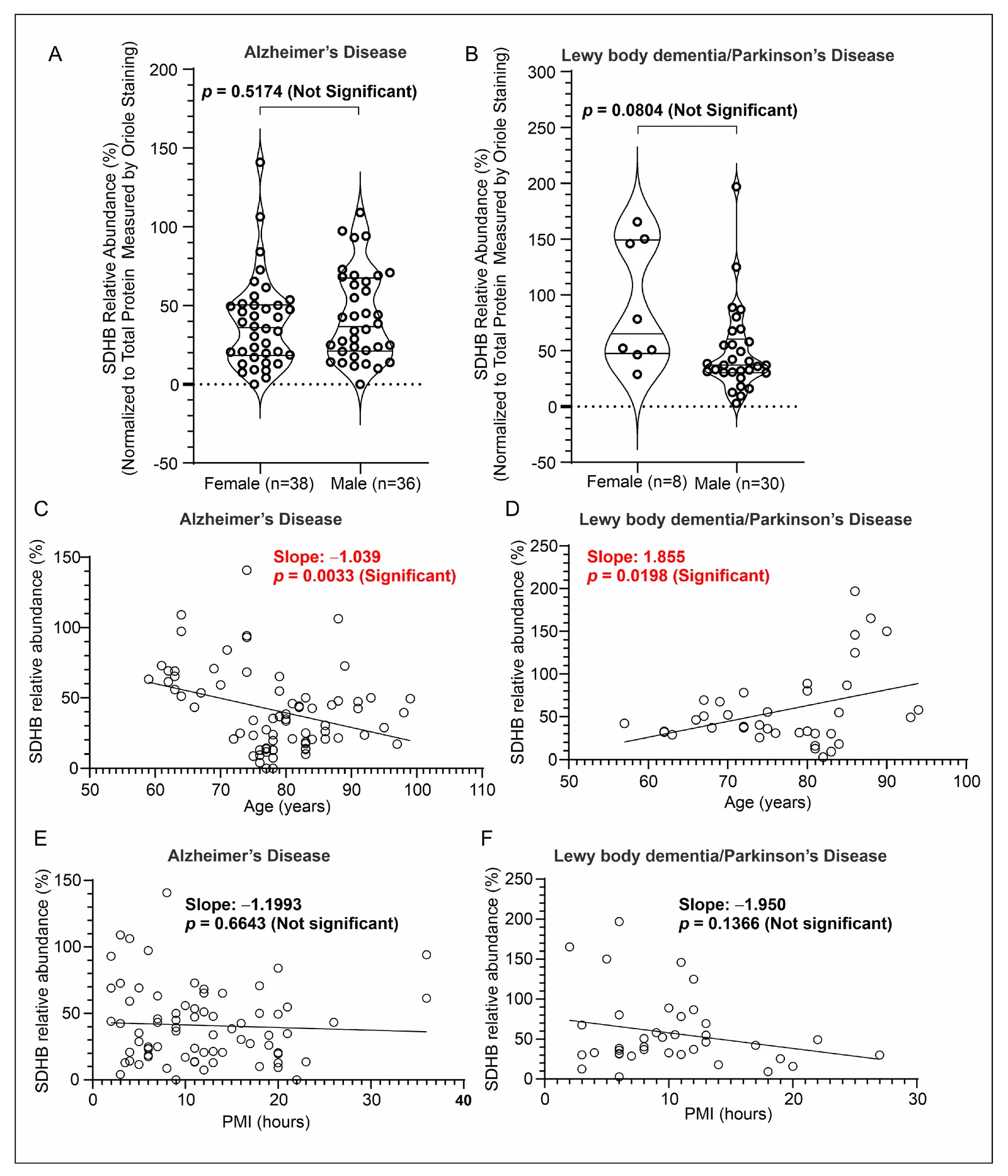
**Associations of SDHB protein abundance with sex, age at death, and postmortem interval in the AD and LBD/PD cohorts.** (**A**,**B**) Violin scatter plots showing temporal cortical SDHB protein abundance in female (*n* = 38) and male (*n* = 36) subjects with AD (**A**) and in female (*n* = 8) and male (*n* = 30) subjects with LBD/PD (**B**). Differences between female and male subjects were analyzed using unpaired Student’s *t*-tests. (**C**,**D**) Scatter plots showing the relationships between age at death and SDHB protein abundance in the AD (**C**) and LBD/PD (**D**) cohorts. (**E**,**F**) Scatter plots showing the relationships between postmortem interval (PMI) and SDHB protein abundance in the AD (**E**) and LBD/PD (**F**) cohorts. Each data point represents one subject. Solid black lines in panels (**C**–**F**) represent simple linear-regression fits generated using GraphPad Prism. The reported *p* values for panels (**C**–**F**) test whether the corresponding regression slopes differ significantly from zero.

**Figure 6:**
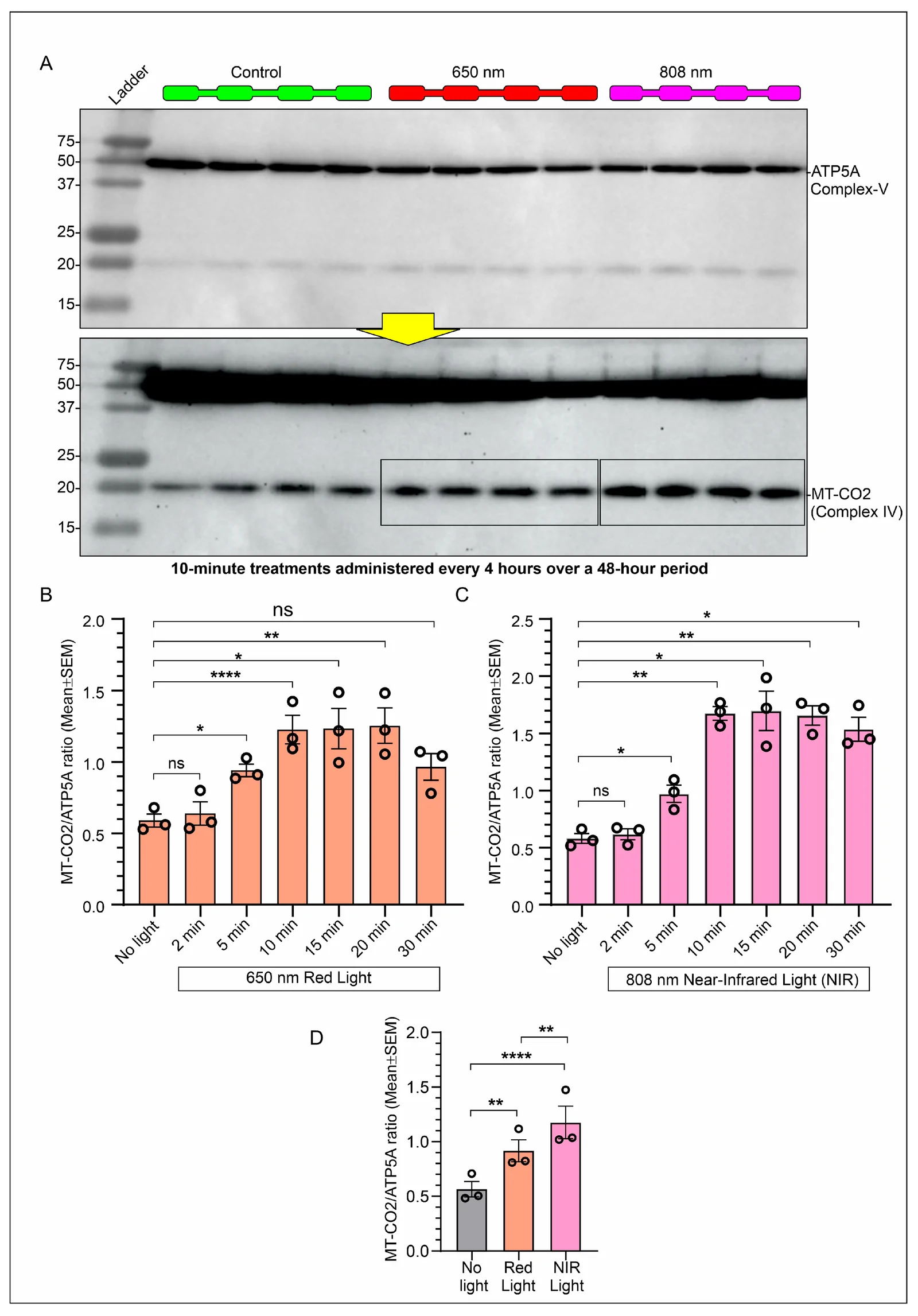
**Effects of 650 nm red and 808 nm NIR light on MT-CO2 protein expression in BE(2)-C neuroblastoma cells.** (**A**) Representative immunoblots showing MT-CO2 and ATP5A protein abundance in BE(2)-C cells following treatment with 650 nm red light or 808 nm near-infrared light for the indicated durations. Cells were exposed to light every 4 h over a 48-h treatment period. Repeated lanes represent technical measurements of the same lysate and were not treated as independent experimental observations. Yellow arrow indicates a higher-exposure image of the same blot. Black rectangles highlight bands exhibiting relatively higher protein abundance. (**B**–**D**) Quantification of MT-CO2 protein abundance. Background-corrected MT-CO2 band intensity was normalized to the corresponding ATP5A band intensity. Values from 3–4 technical replicate lanes were averaged within each condition and independent experiment, and each point represents one experiment-level mean (*n* = 3 independent experiments). Original, untransformed normalized values are displayed. For the exposure-duration analyses in panels (**B**,**C**), experiment-level means were natural-log transformed and analyzed separately using repeated-measures one-way ANOVA followed by Dunnett’s multiple-comparisons test against the matched no-light control. For the matched 10-min exposure experiment in panel (**D**), natural-log-transformed experiment-level means were analyzed using repeated-measures one-way ANOVA followed by Tukey’s multiple-comparisons test for all pairwise comparisons among the no-light, 650-nm red-light, and 808-nm NIR-light conditions. Data are shown as individual experiment-level values with mean ± SEM. Exact adjusted *p* values and experiment-level data are provided in Supplementary Table S4. * *p* < 0.05, ** *p* < 0.01, and **** *p* < 0.0001.

**Figure 7:**
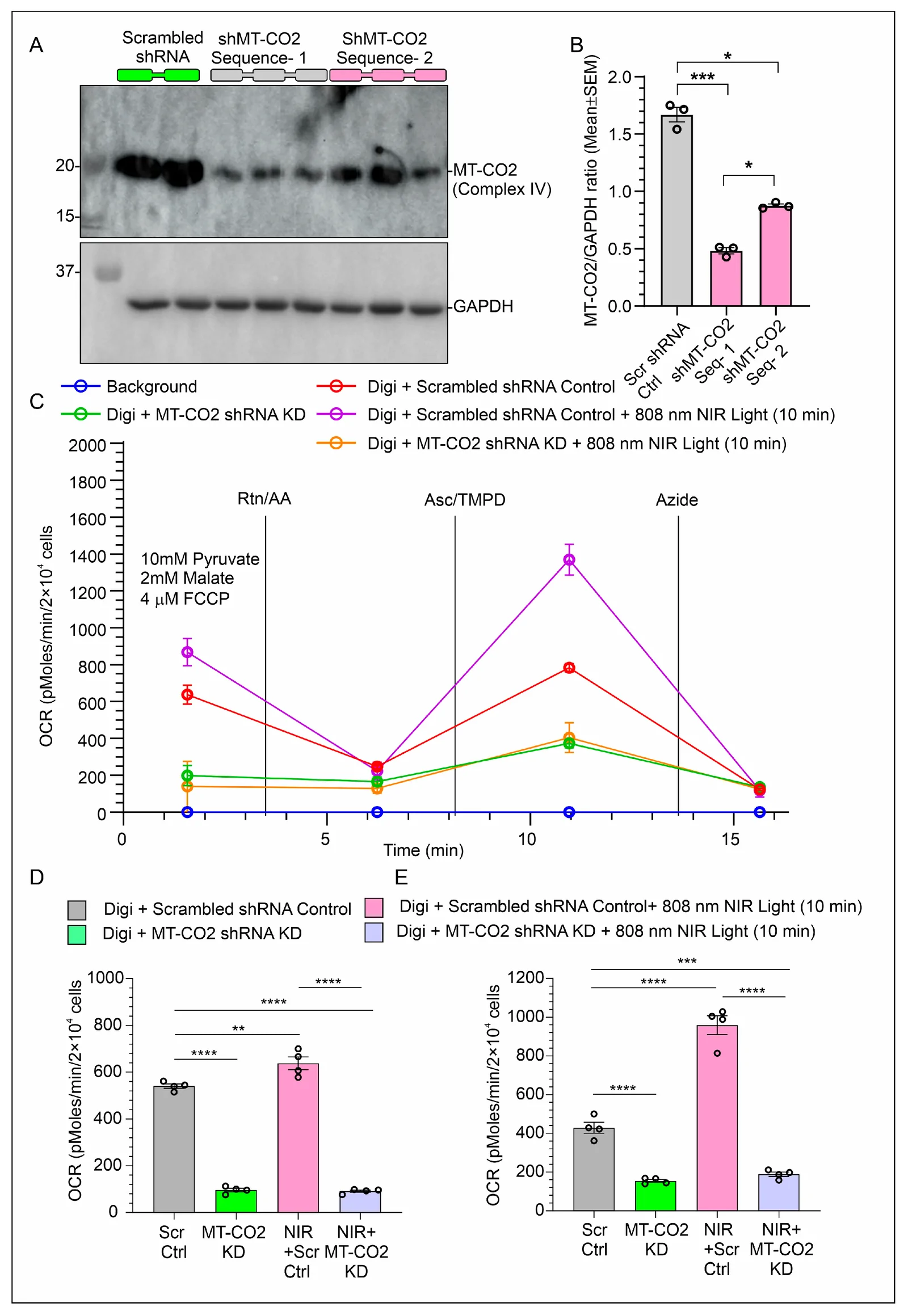
**Effect of MT-CO2 knockdown on 808 nm NIR light-mediated enhancement of mitochondrial respiration in BE(2)-C neuroblastoma cells.** (**A**) Representative immunoblots showing MT-CO2 protein abundance in cells transduced with scrambled shRNA or either of two independent MT-CO2-targeting shRNAs. (**B**) Scatter plot showing MT-CO2 protein abundance. Background-corrected MT-CO2-protein intensity was normalized to the corresponding GAPDH intensity. Technical-replicate values were averaged within each treatment condition and independent experiment, and each point represents one experiment-level mean (*n* = 3 independent experiments). Original, untransformed MT-CO2/GAPDH ratios are displayed. For each protein, experiment-level means were natural-log transformed and analyzed separately using repeated-measures one-way ANOVA, with the Geisser–Greenhouse correction followed by Dunnett’s multiple-comparisons test against the matched scrambled shRNA control. Data are shown as individual experiment-level values with mean ± SEM. Exact adjusted *p* values and experiment-level data are provided in Supplementary Table S5. (**C**) Representative OCR traces showing the mean ± SD of three technical replicate wells per condition from a separate representative experimental run of scramble and MT-CO2 knockdown cells maintained as no-light controls or treated with 808 nm NIR light for 10 min every 4 h over 48 h. This experimental run was not included among the four independent experiments used for the quantitative analyses presented in (**D**,**E**). The traces show the sequential OCR measurements before subtraction of the residual non-mitochondrial/background OCR measured after sodium azide. OCR values were normalized to the measured post-assay cell count and expressed as pmol O_2_/min/2 × 10^4^ cells. Substrate and inhibitor concentrations are provided in the Methods. (**D**,**E**) Quantification of FCCP-stimulated uncoupled Complex I–IV-linked respiration following the addition of pyruvate, malate, and FCCP (**D**), and Complex IV-dependent respiration induced by ascorbate/TMPD after inhibition of Complexes I and III with rotenone and antimycin A (**E**). Residual non-mitochondrial/background OCR measured after sodium azide was subtracted from both the basal uncoupled (FCCP-stimulated) Complex I–IV-linked and Complex IV-dependent measurements presented in (**D**,**E**), respectively. Data were obtained from four independent experiments, with all four conditions assayed on the same day within each experimental run and three technical-replicate wells included per condition. Technical-replicate values were averaged within each condition and experimental run, and each point represents one experiment-level mean. FCCP-stimulated uncoupled Complex I–IV-linked respiration and Complex IV-dependent respiration were analyzed separately using two-way repeated-measures ANOVA, with light treatment and MT-CO2 knockdown status as factors, including their interaction, and matching across light-treatment conditions within each knockdown status (Supplementary Table S5). Šídák’s multiple-comparisons test was used to compare the no-light and 808-nm NIR conditions within each knockdown status, with the two comparisons treated as a single family. Data are shown as individual experiment-level values with mean ± SEM. * *p* < 0.05, ** *p* < 0.01, *** *p* < 0.001 and **** *p* < 0.0001.

**Figure 8:**
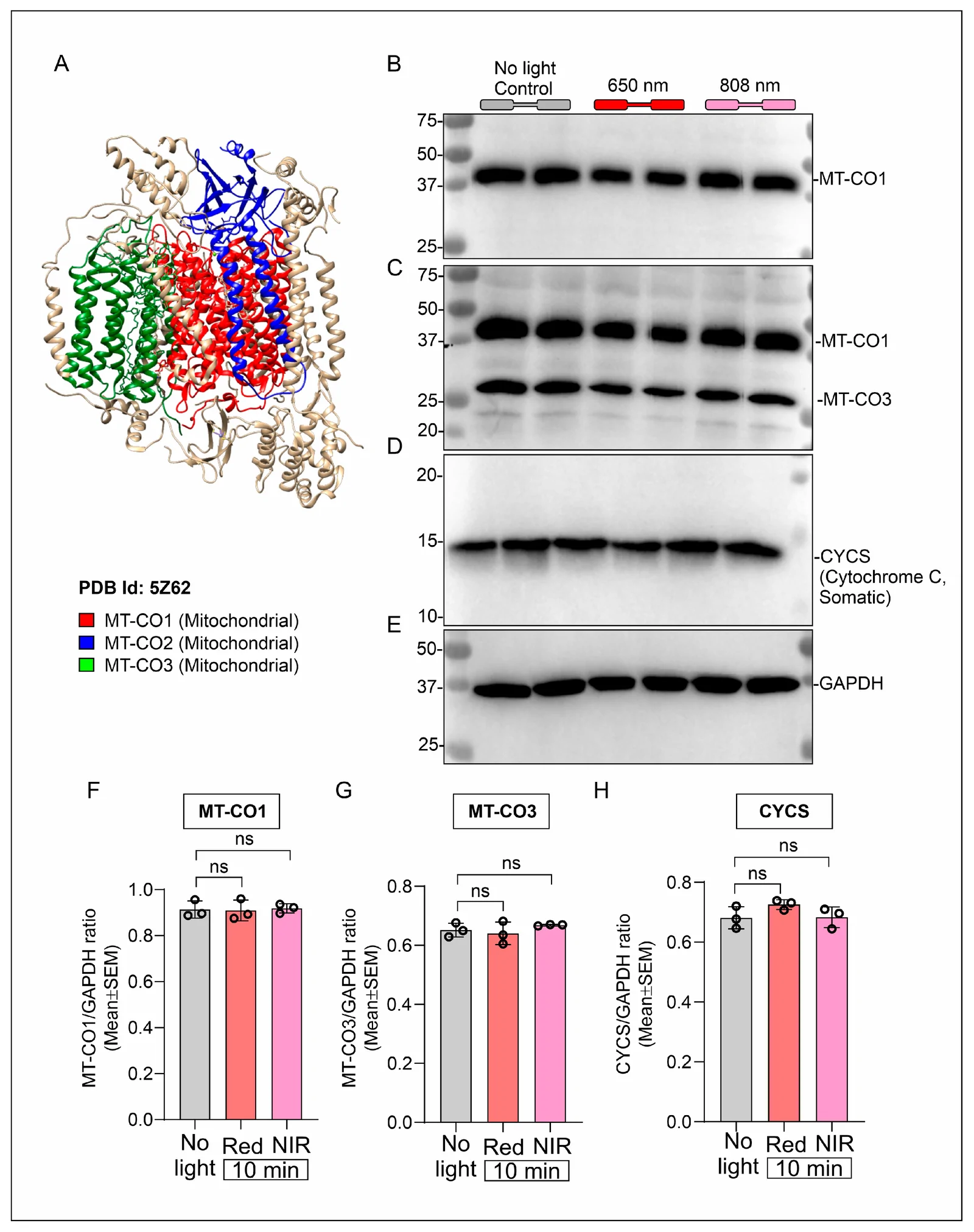
**Effects of 650 nm red and 808 nm near-infrared light on MT-CO1, MT-CO3, and CYCS abundance in BE(2)-C neuroblastoma cells.** (**A**) Structural model of mammalian Complex IV illustrating its multi-subunit organization. The structure was generated using UCSF Chimera software (version 1.17.3) based on Protein Data Bank (PDB) entry 5Z62 [66]. The three mitochondrially encoded core subunits, MT-CO1, MT-CO2, and MT-CO3, are color-coded to highlight their positions within the Complex IV holoenzyme; the remaining subunits are shown in gray. (**B**–**E**) Representative immunoblots showing MT-CO1 (**B**), the MT-CO1 membrane reprobed for MT-CO3 without prior stripping (**C**), CYCS (**D**), and GAPDH (**E**) in BE(2)-C cells maintained as no-light controls or exposed to 650 nm red light or 808 nm NIR light. Cells were exposed to light for 10 min every 4 h over a 48-h period. The yellow arrow indicates that the MT-CO1 blot was subsequently reprobed with the anti-MT-CO3 antibody without prior stripping of the membrane. GAPDH was detected on separate membranes containing aliquots of the same lysates arranged in the corresponding lane order. (**F**–**H**) Quantification of MT-CO1 (**F**), MT-CO3 (**G**), and CYCS (**H**) abundance. Background-corrected target-protein intensity was normalized to the corresponding GAPDH intensity. Technical-replicate values were averaged within each treatment condition and independent experiment, and each point represents one experiment-level mean (*n* = 3 independent experiments). Original, untransformed target-protein/GAPDH ratios are displayed. For each target protein, experiment-level means were natural-log transformed and analyzed separately using repeated-measures one-way ANOVA, implemented in GraphPad Prism, with the Geisser–Greenhouse correction followed by Dunnett’s multiple-comparisons test against the matched no-light control. Data are shown as individual experiment-level values with mean ± SEM. Exact adjusted *p* values and experiment-level data are provided in Supplementary Table S6. ns: not significant.

## Data Availability

The raw data supporting the conclusions of this article will be made available by the authors on request.

## Supplementary Materials

The following supporting information can be downloaded at: https://www.mdpi.com/article/10.3390/cells15211941/s1, Figure S1. ImageJ-based separation and quantification of the closely migrating MT-CO2 and NDUFB8 immunoreactive signals. (A) Representative OXPHOS-cocktail immunoblot showing MT-CO2 abundance in temporal cortex samples from CN reference sample (A21-006) and LBD/PD subjects. This blot, also presented in Figure 2A, was selected as an example because the MT-CO2 and lower-migrating NDUFB8 signals are closely spaced in several lanes. The red dotted rectangles in panels (A–C) identify regions in which the MT-CO2 and NDUFB8 signals migrated in close proximity. Representative intensity profiles from these regions are shown in panel (D) to illustrate how the MT-CO2 signal was distinguished from and quantified separately from the adjacent NDUFB8 signal. (B) Quantification-exposure image of the same immunoblot shown in panel (A). This exposure was used to maintain the immunoreactive signals within the quantitative detection range and improve discrimination between the MT-CO2 and NDUFB8 signals. The red arrow indicates that panels (A) and (B) show different exposures of the same immunoblot. (C) ImageJ lane selections used for densitometric analysis. Rectangular selections of consistent width were positioned over individual lanes to encompass the MT-CO2 and NDUFB8 signals together with the adjacent local background. The lane selections are outlined in yellow and numbered 1–24. The SDHB signal detected by the OXPHOS antibody cocktail is also visible above MT-CO2. (D) Representative intensity-profile plots from lanes 1–5 and 22–24, corresponding to the regions outlined by the red dotted rectangles in panels (A–C). The principal peak corresponds to MT-CO2, whereas the lower-migrating NDUFB8 signal appears as a shoulder or separate adjacent peak. The boundary between the MT-CO2 and NDUFB8 components was manually defined at the intervening minimum or inflection point in the intensity profile, as indicated by the red lines. Blue arrows identify the NDUFB8-associated component excluded from MT-CO2 quantification. The yellow-filled area represents the integrated MT-CO2 peak area measured using the ImageJ Wand (Tracing) tool after background correction. The individual intensity profiles were exported separately and arranged to fit the figure dimensions; therefore, the profiles illustrate the peak-separation procedure but are not displayed on a common numerical scale; Table S1. Statistical comparisons of MT-CO2 protein abundance among diagnostic groups corresponding to Figure 2C. The table provides sample sizes, mean differences, 95% confidence intervals, and Tukey-adjusted *p* values for all pairwise comparisons among CN, AD, LBD/PD, and PSP groups; Table S2. Statistical comparisons of SDHB protein abundance among diagnostic groups corresponding to Figure 4B. The table provides sample sizes, mean differences, 95% confidence intervals, and Tukey-adjusted *p* values for all pairwise comparisons among CN, AD, LBD/PD, and PSP groups; Table S3. Two-way repeated-measures ANOVA and Šídák-adjusted comparisons of MT-CO2 and SDHB protein abundance across diagnostic groups. Normalized MT-CO2 and SDHB abundance values from CN (*n* = 9), Alzheimer’s disease subjects (AD; *n* = 74), Lewy body dementia/Parkinson’s disease subjects (LBD/PD; *n* = 38), and progressive supranuclear palsy subjects (PSP; *n* = 11) were analyzed using two-way repeated-measures ANOVA, with protein and diagnosis as factors. MT-CO2 and SDHB values were matched within subjects. Šídák’s multiple-comparisons test was used for pairwise diagnostic-group comparisons within each protein, with all 12 comparisons included in a single family. Mean differences were calculated as the first diagnostic group minus the second diagnostic group. Adjusted *p* values < 0.05 were considered statistically significant; Table S4. Experiment-level data and statistical results corresponding to Figure 6B–D. The table presents normalized MT-CO2/ATP5A ratios, log-transformed values used for analysis, and exact adjusted p values for the red-light and NIR-light treatment comparisons; Table S5. Experiment-level data and statistical analyses corresponding to Figure 7B,D,E. The table presents normalized MT-CO2/GAPDH ratios, mitochondrial respiration measurements, transformed values used for analysis, and exact adjusted *p* values for the MT-CO2-knockdown and NIR-light treatment analyses; Table S6. Experiment-level data and statistical analyses corresponding to Figure 8F–H. The table presents normalized MT-CO1/GAPDH, MT-CO3/GAPDH, and CYCS/GAPDH ratios, log-transformed values used for analysis, and exact Dunnett-adjusted *p* values for the red-light and NIR-light treatment comparisons.
